# Supplementary material for: Novel NIR-Phosphorescent Ir(III) Complexes: Synthesis, Characterization and Their Exploration as Lifetime-Based O2 Sensors in Living Cells
Source: Molecules. 2022 May 14;27(10):3156. doi: 10.3390/molecules27103156 (PMC9144934; doi:10.3390/molecules27103156)
Supplement: Supplementary file 1 [file molecules-27-03156-s001.zip › molecules-1713432-supplementary.pdf]

**Electronic Supporting Information**

**for the article**

# Novel NIR-phosphorescent Ir(III) complexes: synthesis, characterization and their exploration as lifetime-based $O_2$ sensors in living cells

*Ilya S. Kritchenkov[1], Vitaliya G. Mikhnevich[1], Victoria S. Stashchak[1], Anastasia I. Solomatina[1], Daria O. Kozina[1], Victor V. Sokolov[1] and Sergey P. Tunik[1*]*

*[1]Saint-Petersburg State University, Institute of Chemistry, Universitetskii pr., 26, St. Petersburg, 198504, Russian Federation*

## Content

NMR spectra of complexes **1-7** **30**

HRMS ESI spectra of complexes **1-7** **38**

UV-vis absorption spectra of complexes **1-7** **42**

DFT calculations of complexes **1-7** **43**

Phosphorescence decay curves of complexes **1** and **7** **50**

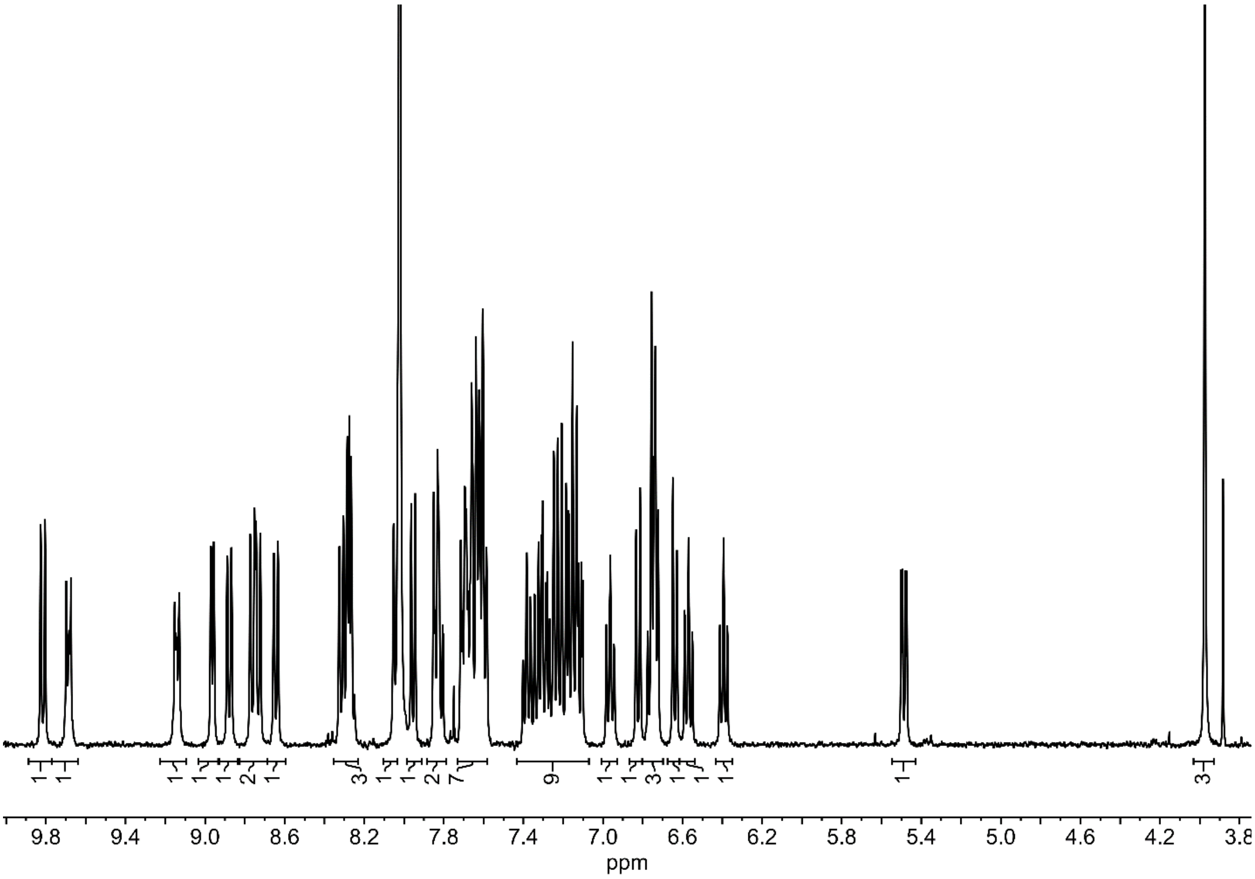


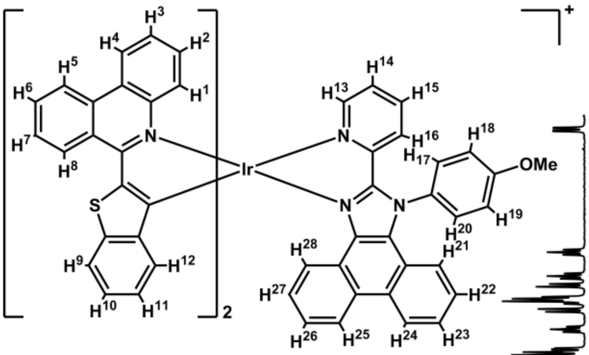


| | | |
|---|---|---|
| $H^{1}$ = 9.82 | $H^{1'}$ = 7.19 | $H^{13}$ = 8.97 |
| $H^{2}$ = 7.15 | $H^{2'}$ = 6.96 | $H^{14}$ = 7.62 |
| $H^{3}$ = 7.60 | $H^{3'}$ = 7.68 | $H^{15}$ = 7.83 |
| $H^{4}$ = 8.88 | $H^{4'}$ = 8.65 | $H^{16}$ = 6.73 |
| $H^{5}$ = 9.14 | $H^{5'}$ = 8.32 | $H^{17}$ = 7.64 |
| $H^{6}$ = 8.28 | $H^{6'}$ = 7.38 | $H^{18}$ = 7.28 |
| $H^{7}$ = 8.26 | $H^{7'}$ = 7.32 | $H^{19}$ = 7.11 |
| $H^{8}$ = 9.69 | $H^{8'}$ = 7.70 | $H^{20}$ = 5.48 |
| $H^{9}$ = 7.95 | $H^{9'}$ = 7.83 | $H^{21}$ = 6.74 |
| $H^{10}$ = 7.15 | $H^{10'}$ = 7.22 | $H^{22}$ = 7.24 |
| $H^{11}$ = 6.57 | $H^{11'}$ = 6.75 | $H^{23}$ = 7.65 |
| $H^{12}$ = 6.82 | $H^{12'}$ = 6.64 | $H^{24}$ = 8.73 |
| | | $H^{25}$ = 8.77 |
| | | $H^{26}$ = 7.61 |
| | | $H^{27}$ = 6.40 |
| | | $H^{28}$ = 8.05 |

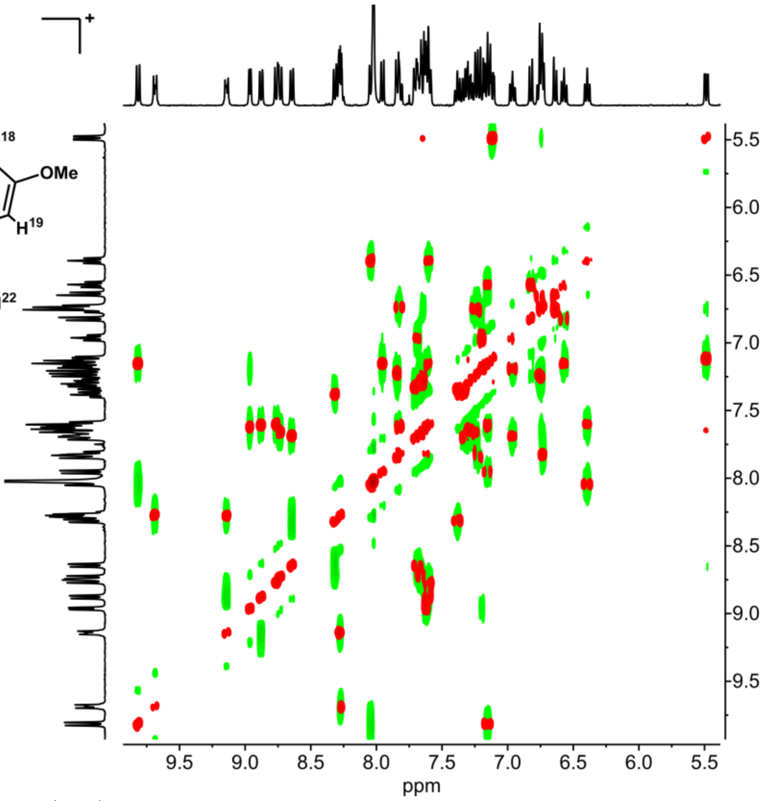


Figure S1. 1D $^{1}H$ NMR (top) and 2D $^{1}H$-$^{1}H$ COSY and NOESY (bottom) NMR spectra of complex **1**. Solution in $(CD_3)_2CO$, temperature 298 K. Red signals are for COSY spectrum, green signals are for NOESY spectrum.

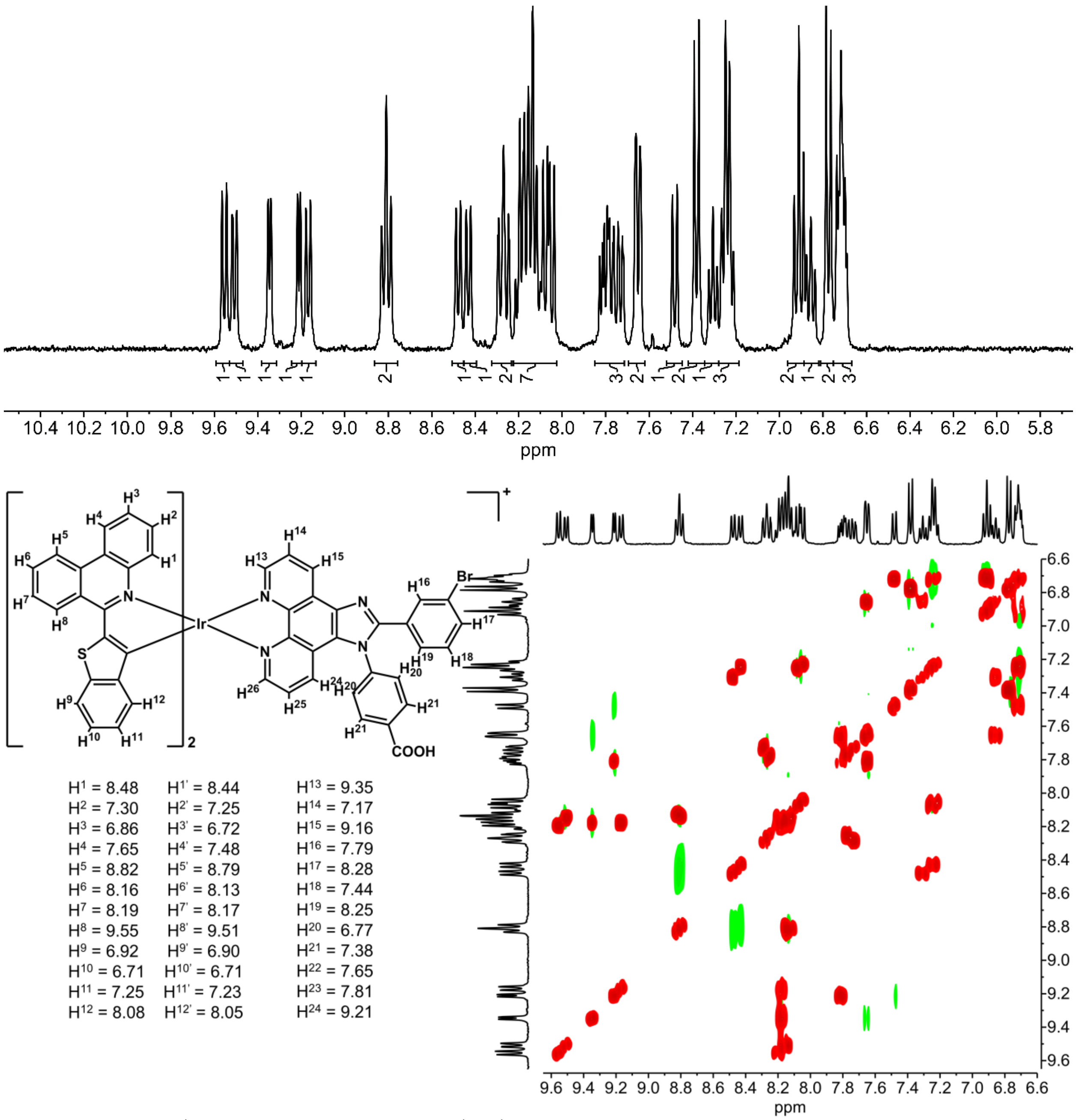


Figure S2. 1D $^{1}H$ NMR (top) and 2D $^{1}H$-$^{1}H$ COSY and NOESY (bottom) NMR spectra of complex **2**. Solution in $(CD_3)_2CO$, temperature 298 K. Red signals are for COSY spectrum, green signals are for NOESY spectrum.

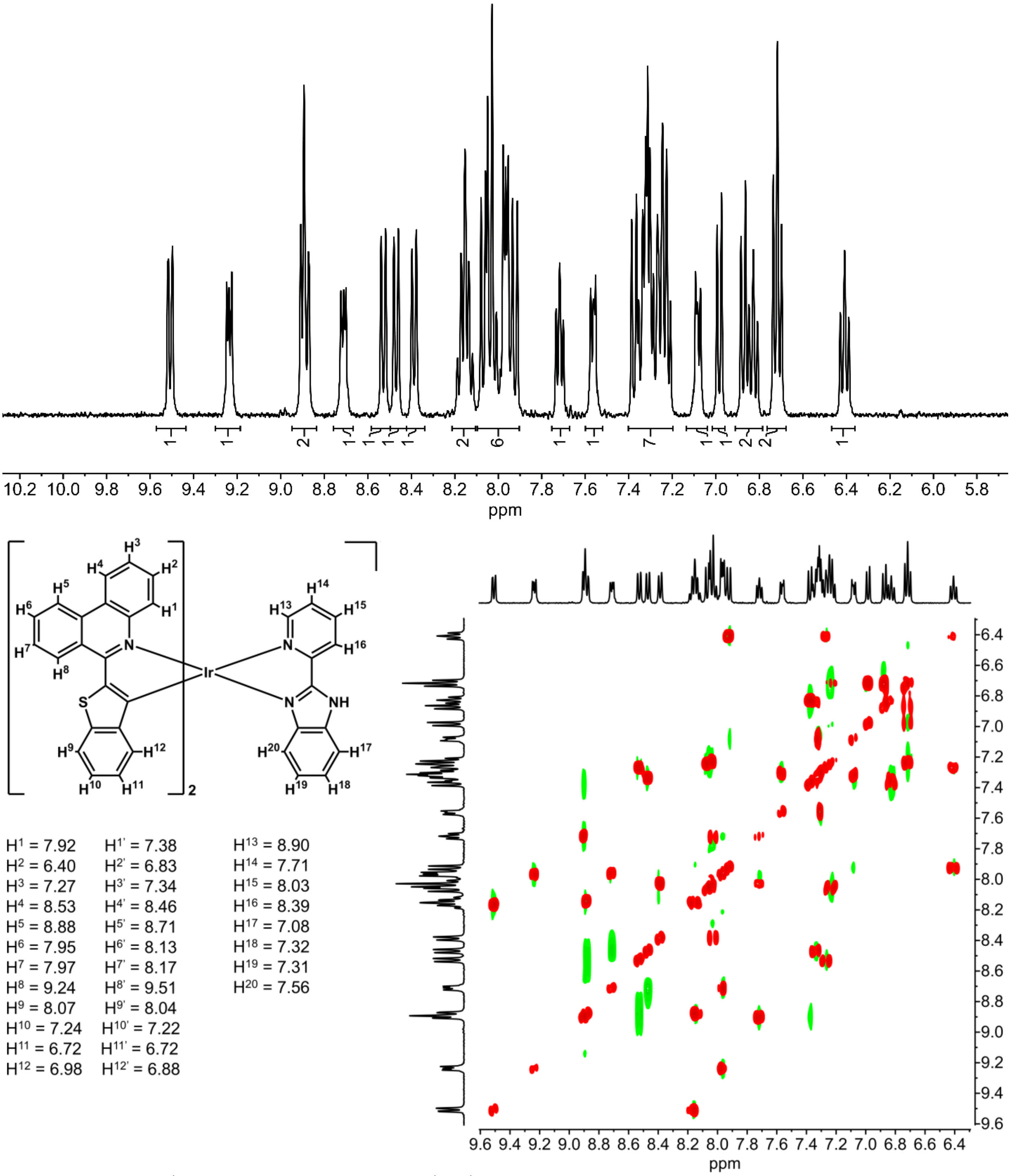


Figure S3. 1D $^{1}H$ NMR (top) and 2D $^{1}H$-$^{1}H$ COSY and NOESY (bottom) NMR spectra of complex **3**. Solution in $(CD_3)_2CO$, temperature 298 K. Red signals are for COSY spectrum, green signals are for NOESY spectrum.

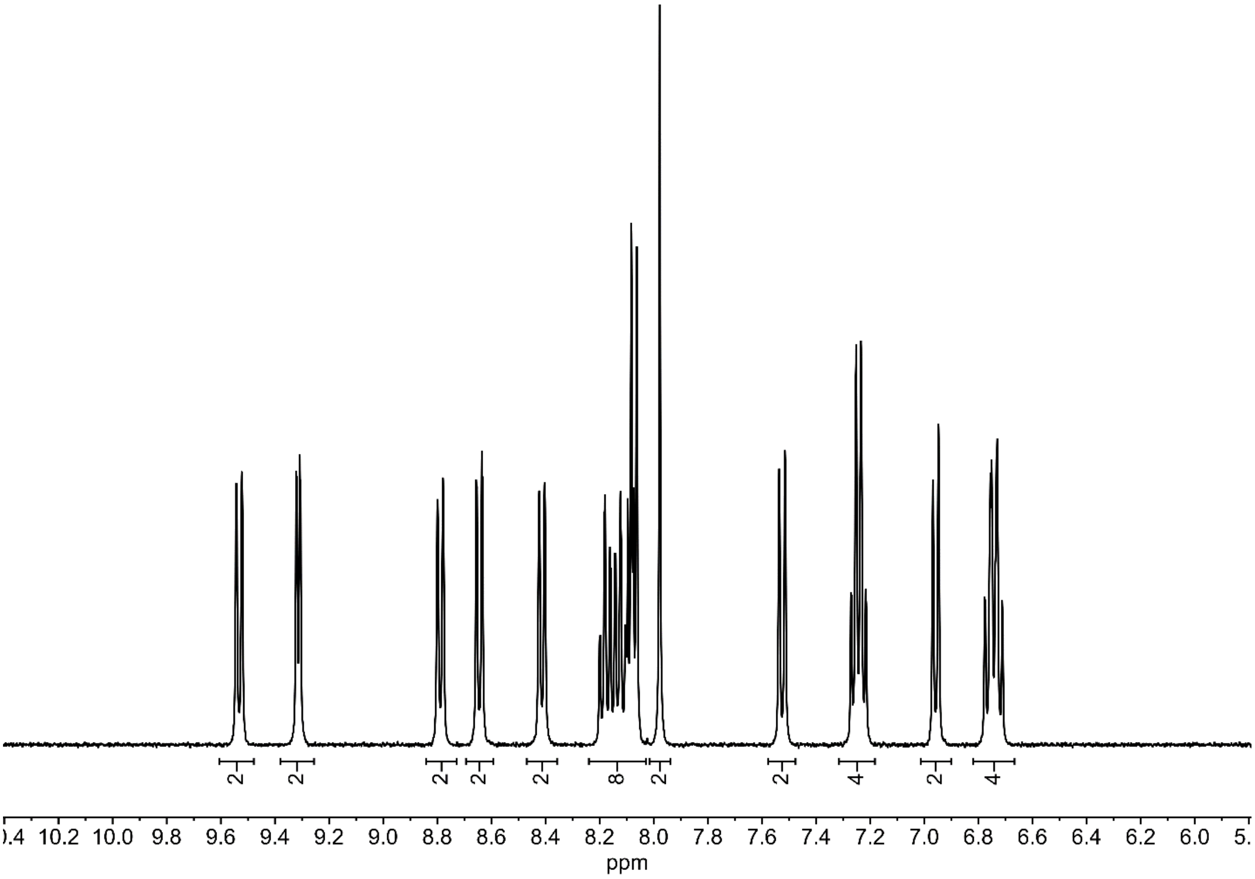

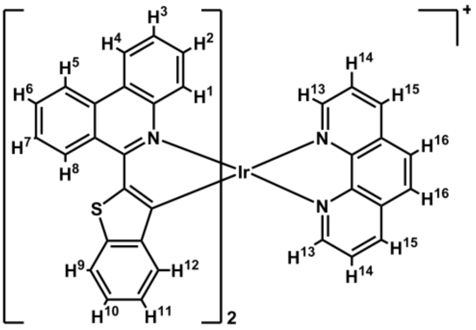

H¹ = 7.52
H² = 6.75
H³ = 7.23
H⁴ = 8.41
H⁵ = 8.79
H⁶ = 8.12
H⁷ = 8.18
H⁸ = 9.53
H⁹ = 8.07
H¹⁰ = 7.25
H¹¹ = 6.73
H¹² = 6.96

H¹³ = 9.31
H¹⁴ = 8.08
H¹⁵ = 8.64
H¹⁶ = 7.98

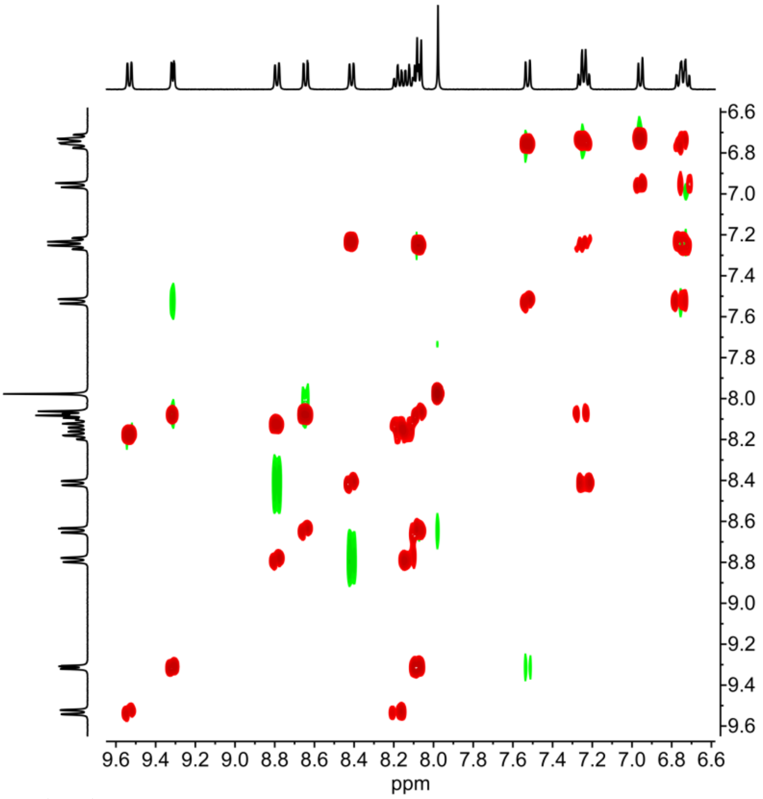

Figure S4. 1D $^{1}$H NMR (top) and 2D $^{1}$H-$^{1}$H COSY and NOESY (bottom) NMR spectra of complex **4**. Solution in $(CD_3)_2CO$, temperature 298 K. Red signals are for COSY spectrum, green signals are for NOESY spectrum.

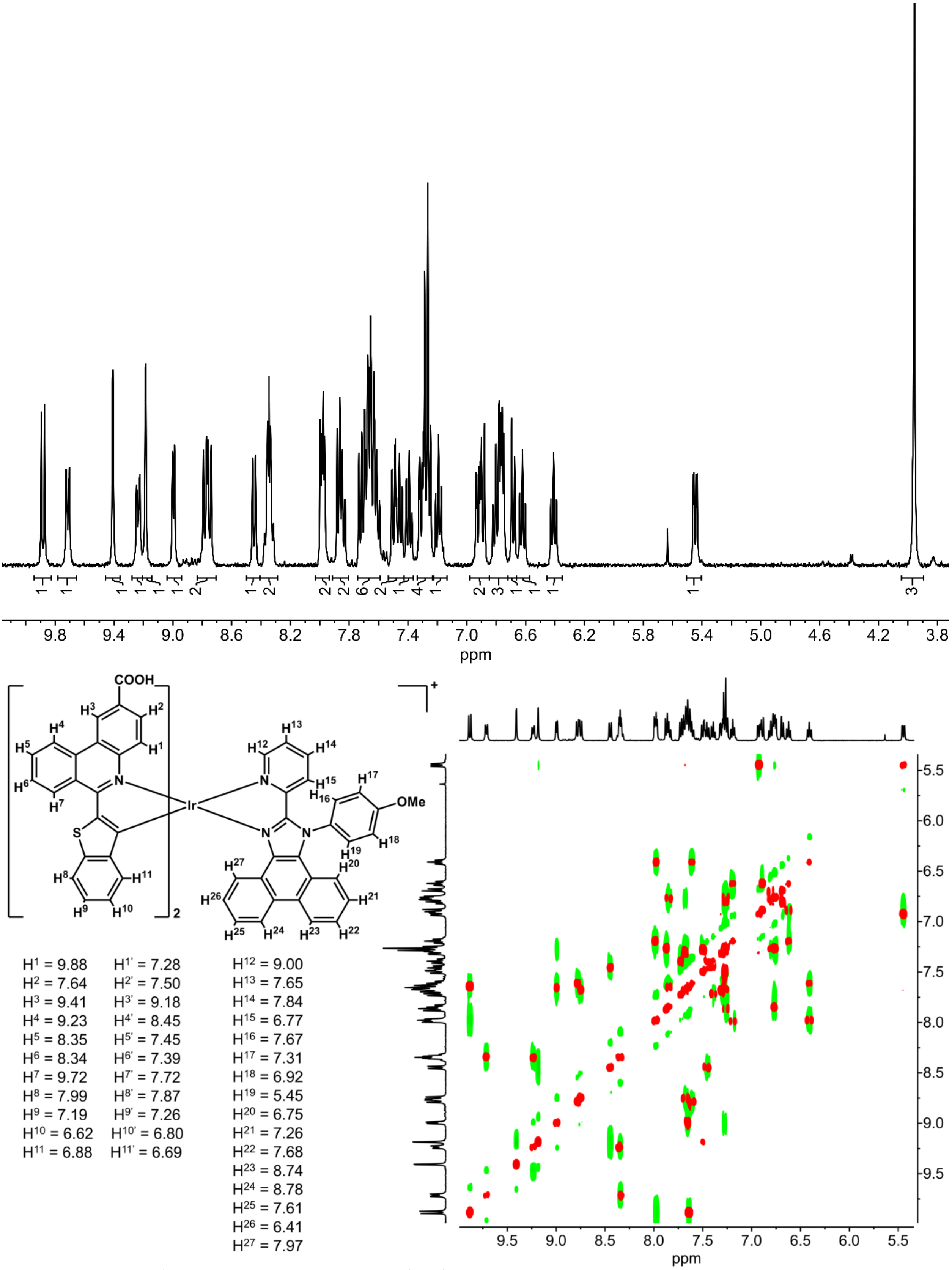


Figure S5. 1D $^{1}H$ NMR (top) and 2D $^{1}H$-$^{1}H$ COSY and NOESY (bottom) NMR spectra of complex **5**. Solution in $(CD_3)_2CO$, temperature 298 K. Red signals are for COSY spectrum, green signals are for NOESY spectrum.

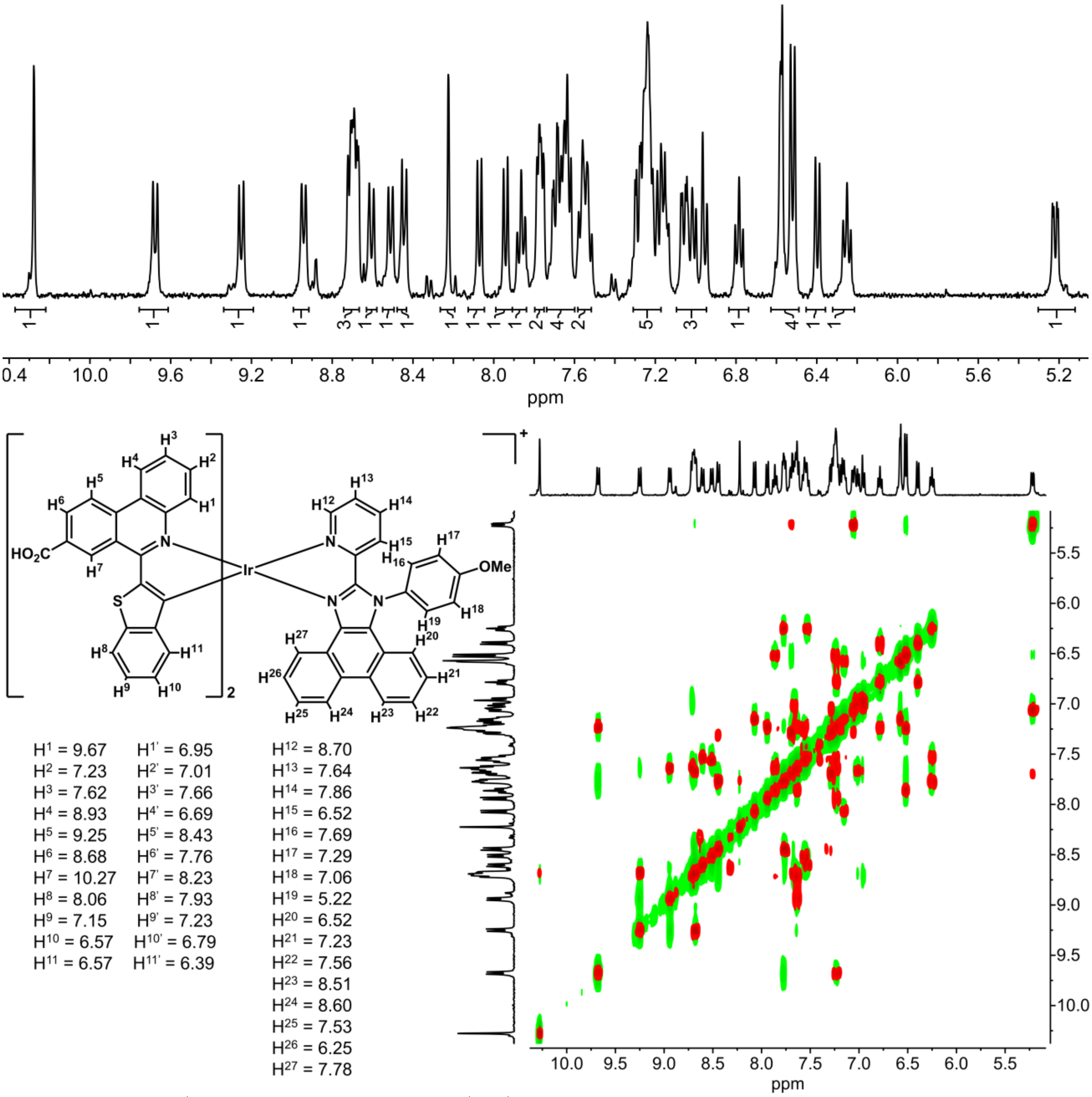


Figure S6. 1D $^{1}H$ NMR (top) and 2D $^{1}H$-$^{1}H$ COSY and NOESY (bottom) NMR spectra of complex **6**. Solution in $(CD_3)_2CO$, temperature 298 K. Red signals are for COSY spectrum, green signals are for NOESY spectrum.

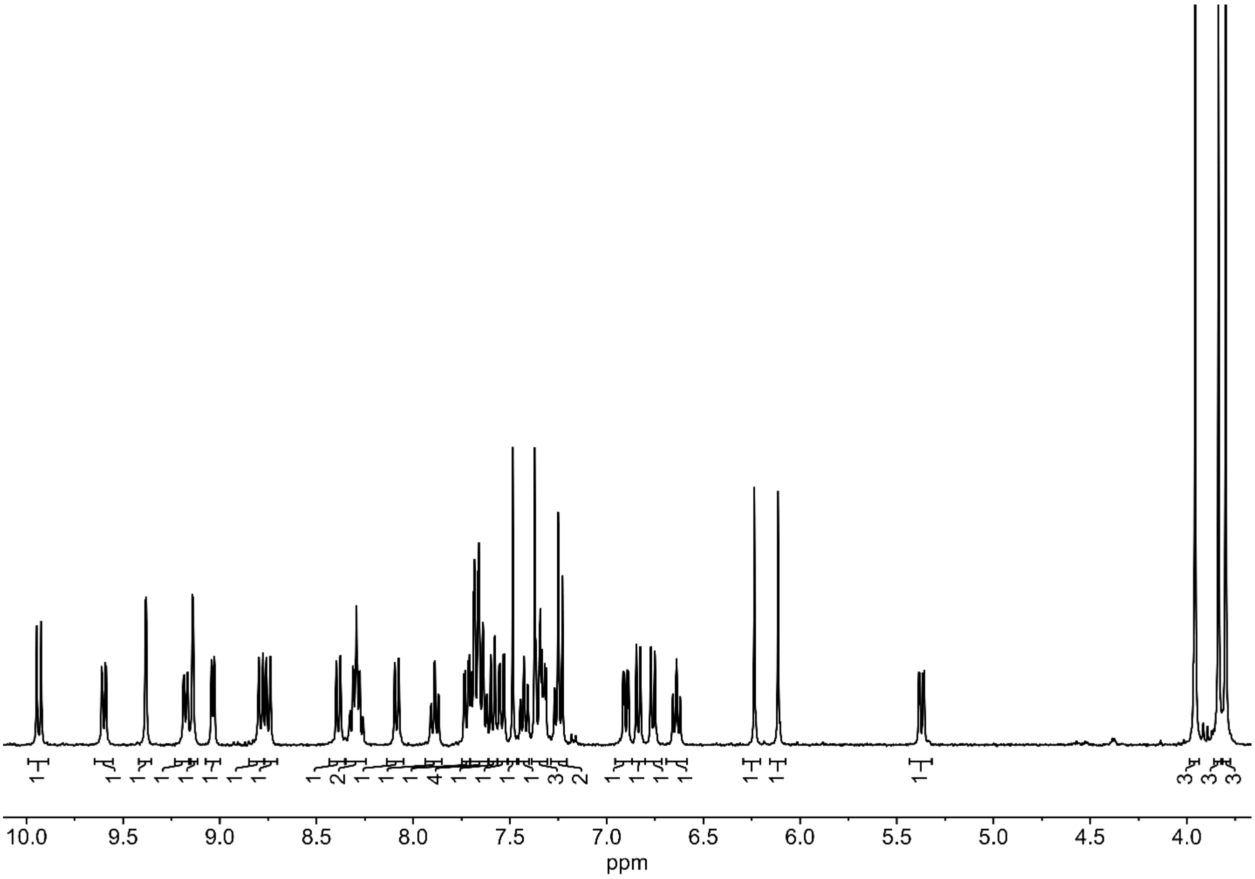


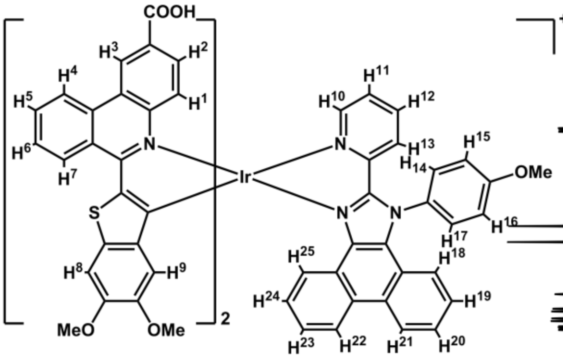


| | | |
|---|---|---|
| $H^1$ = 9.93 | $H^{1'}$ = 7.24 | $H^{10}$ = 9.04 |
| $H^2$ = 7.67 | $H^{2'}$ = 7.54 | $H^{11}$ = 7.68 |
| $H^3$ = 9.38 | $H^{3'}$ = 9.14 | $H^{12}$ = 7.89 |
| $H^4$ = 9.18 | $H^{4'}$ = 8.38 | $H^{13}$ = 6.84 |
| $H^5$ = 8.31 | $H^{5'}$ = 7.43 | $H^{14}$ = 7.72 |
| $H^6$ = 8.28 | $H^{6'}$ = 7.34 | $H^{15}$ = 7.32 |
| $H^7$ = 9.60 | $H^{7'}$ = 7.59 | $H^{16}$ = 6.90 |
| $H^8$ = 7.49 | $H^{8'}$ = 7.37 | $H^{17}$ = 5.37 |
| $H^9$ = 6.23 | $H^{9'}$ = 6.11 | $H^{18}$ = 6.76 |
| | | $H^{19}$ = 7.25 |
| | | $H^{20}$ = 7.65 |
| | | $H^{21}$ = 8.74 |
| | | $H^{22}$ = 8.79 |
| | | $H^{23}$ = 7.64 |
| | | $H^{24}$ = 6.63 |
| | | $H^{25}$ = 8.08 |

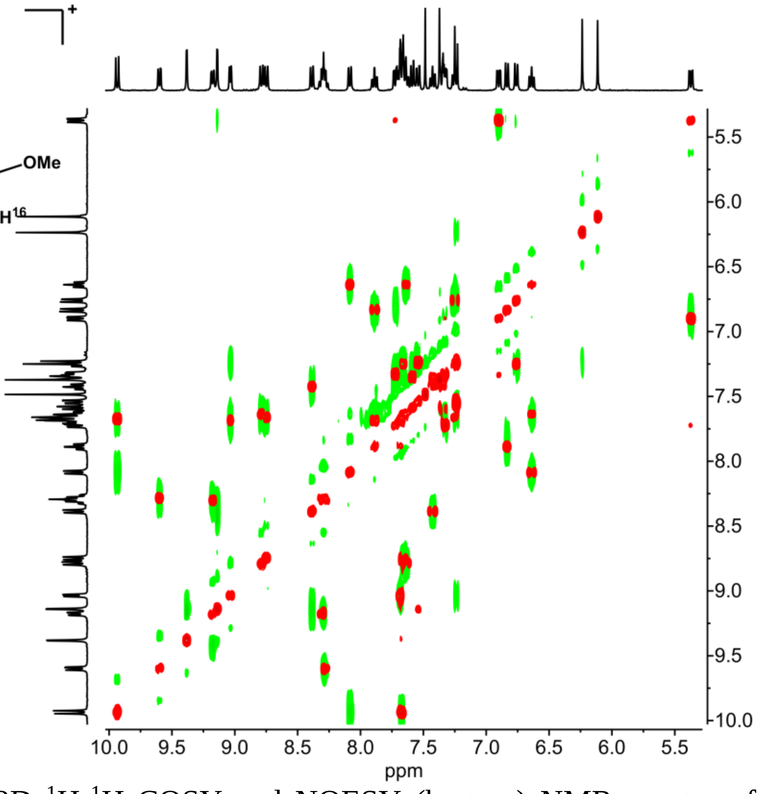


Figure S7a. 1D $^1H$ NMR (top) and 2D $^1H$-$^1H$ COSY and NOESY (bottom) NMR spectra of complex **7**. Solution in $(CD_3)_2CO$, temperature 298 K. Red signals are for COSY spectrum, green signals are for NOESY spectrum.

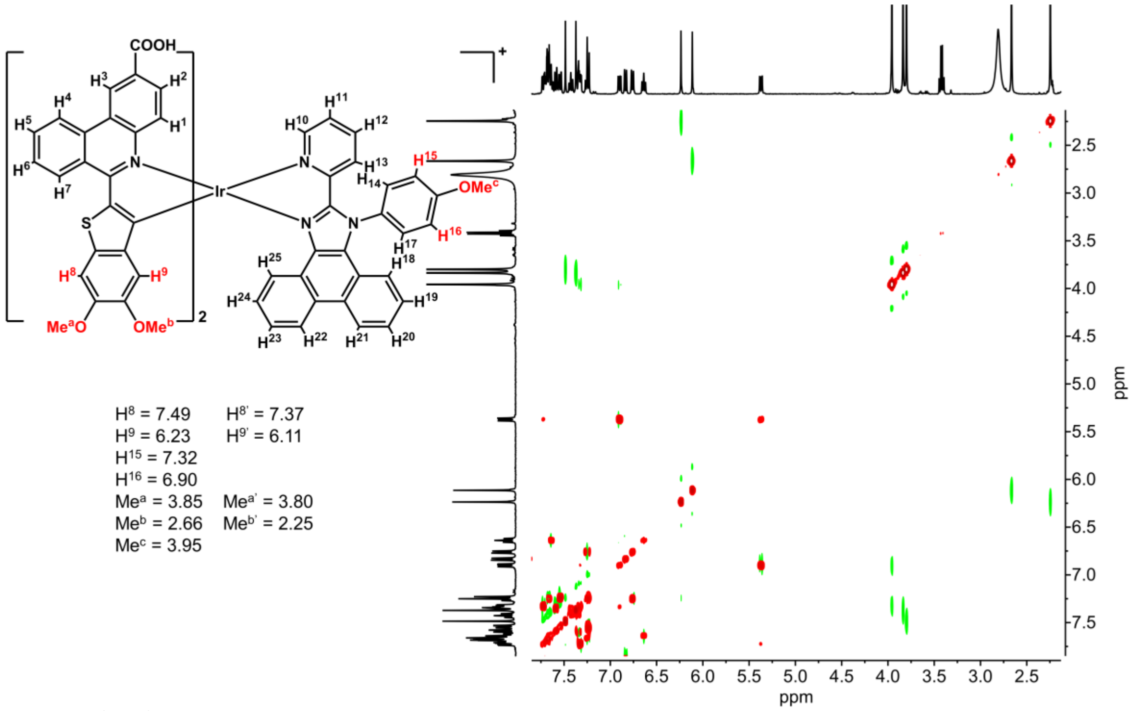


Figure S7b. 2D $^{1}H$-$^{1}H$ COSY and NOESY (bottom) NMR spectra of complex **7** (interactions with aliphatic part). Solution in $(CD_3)_2CO$, temperature 298 K. Red signals are for COSY spectrum, green signals are for NOESY spectrum.

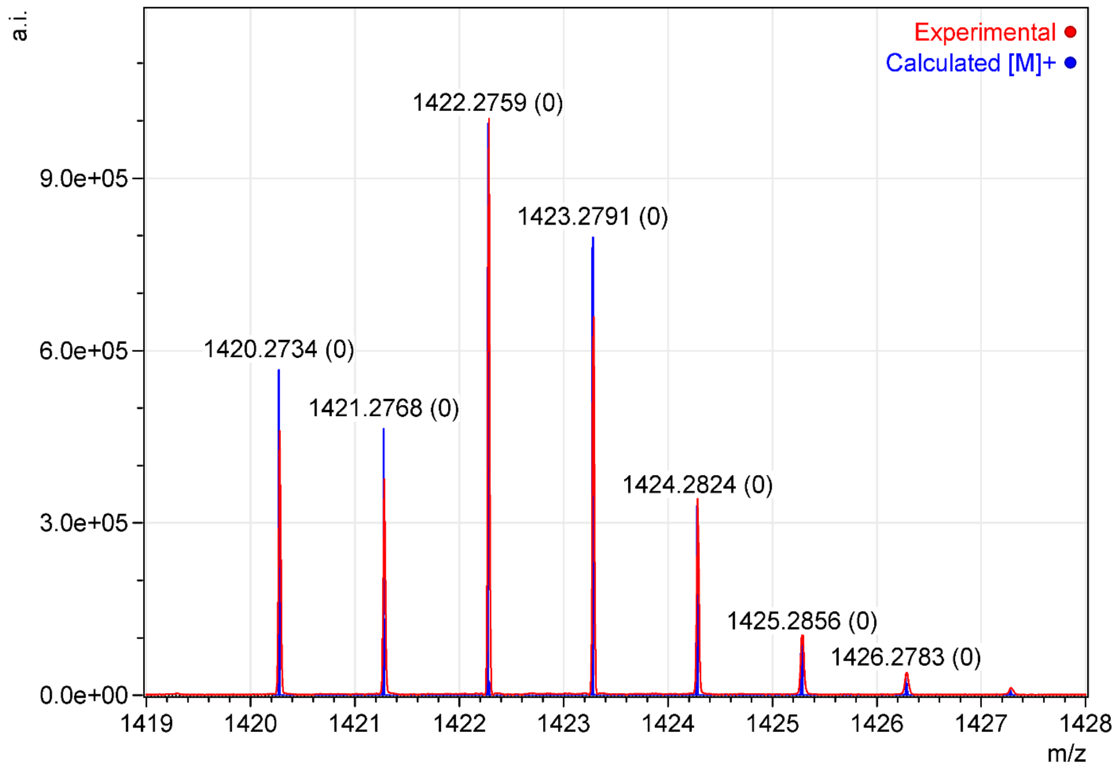


Figure S8. Fragment of experimental and simulated HR-ESI mass-spectra of complex **1**. Solution in methanol.

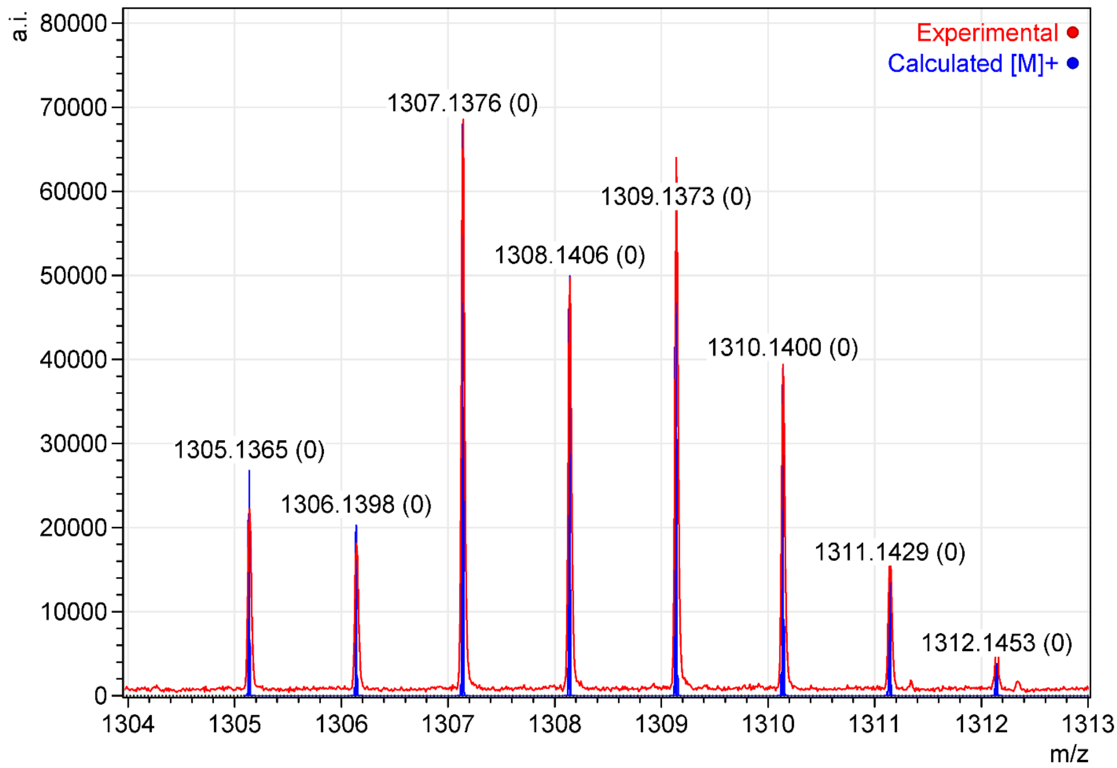


Figure S9. Fragment of experimental and simulated HR-ESI mass-spectra of complex **2**. Solution in methanol.

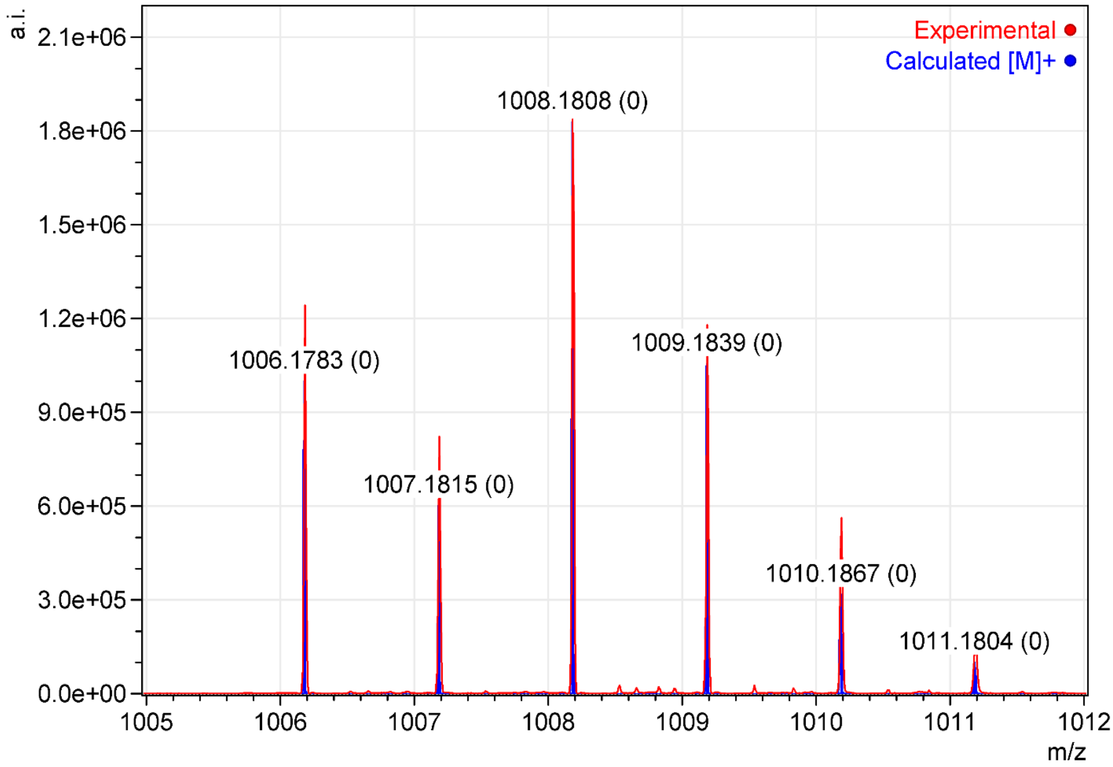


Figure S10. Fragment of experimental and simulated HR-ESI mass-spectra of complex **3**. Solution in methanol.

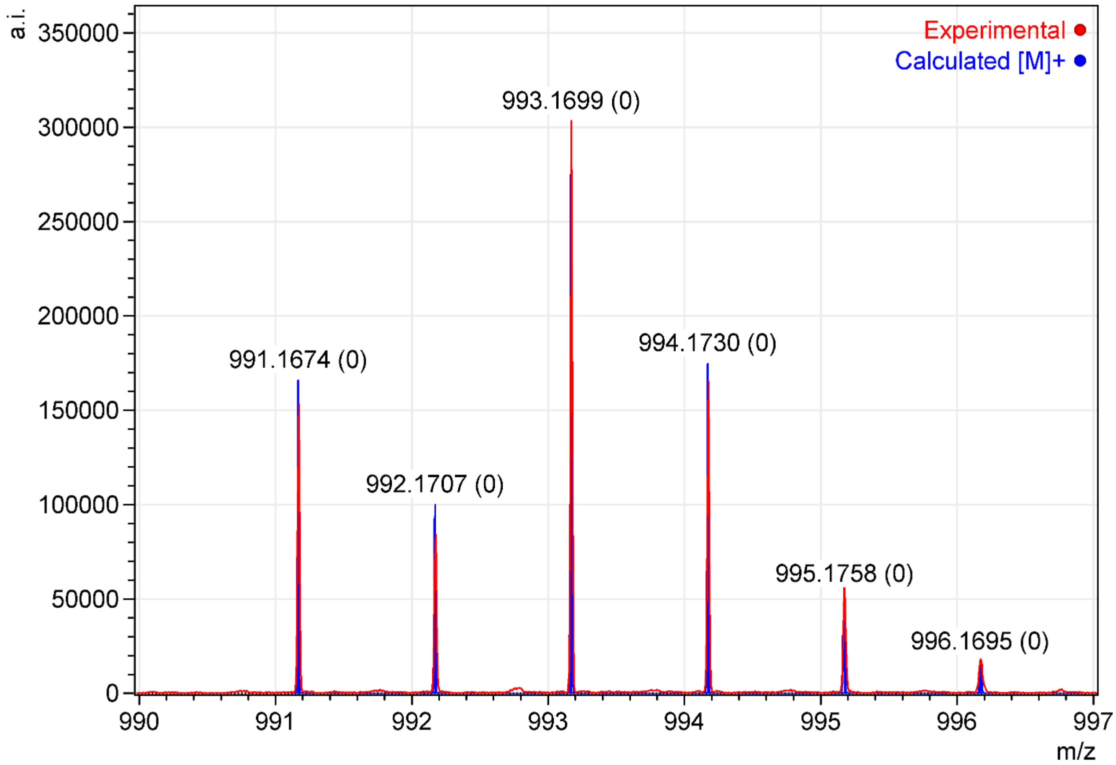


Figure S11. Fragment of experimental and simulated HR-ESI mass-spectra of complex **4**. Solution in methanol.

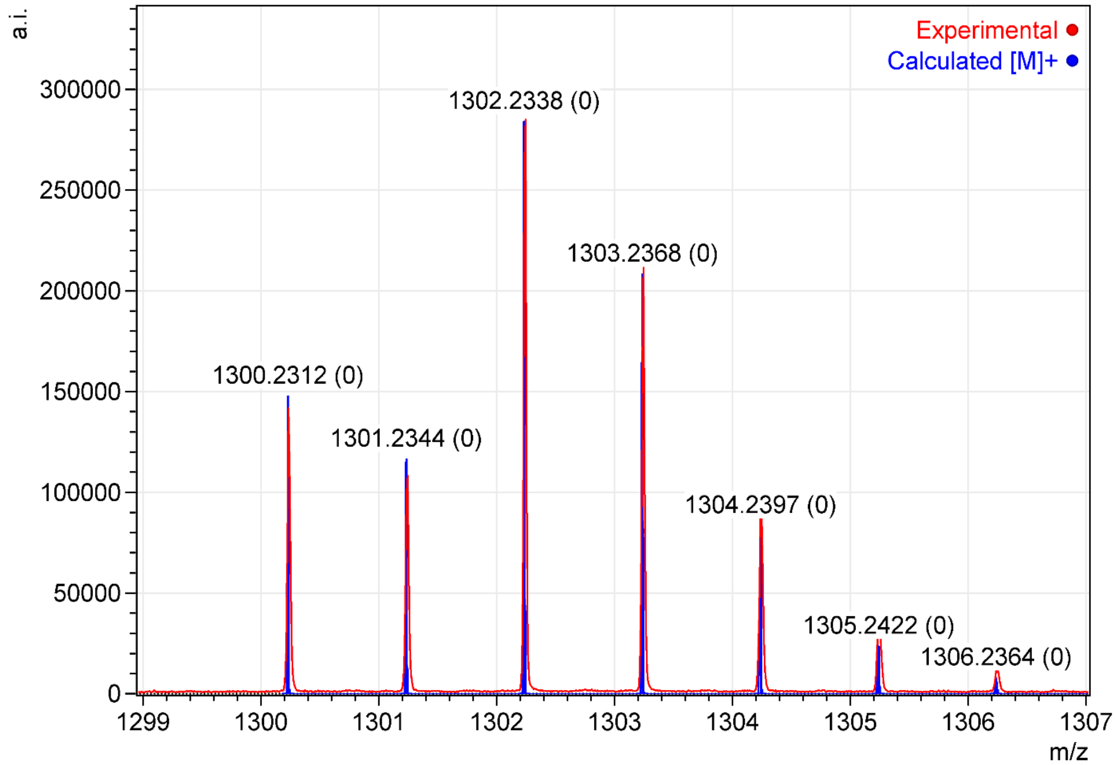


Figure S12. Fragment of experimental and simulated HR-ESI mass-spectra of complex **5**. Solution in methanol.

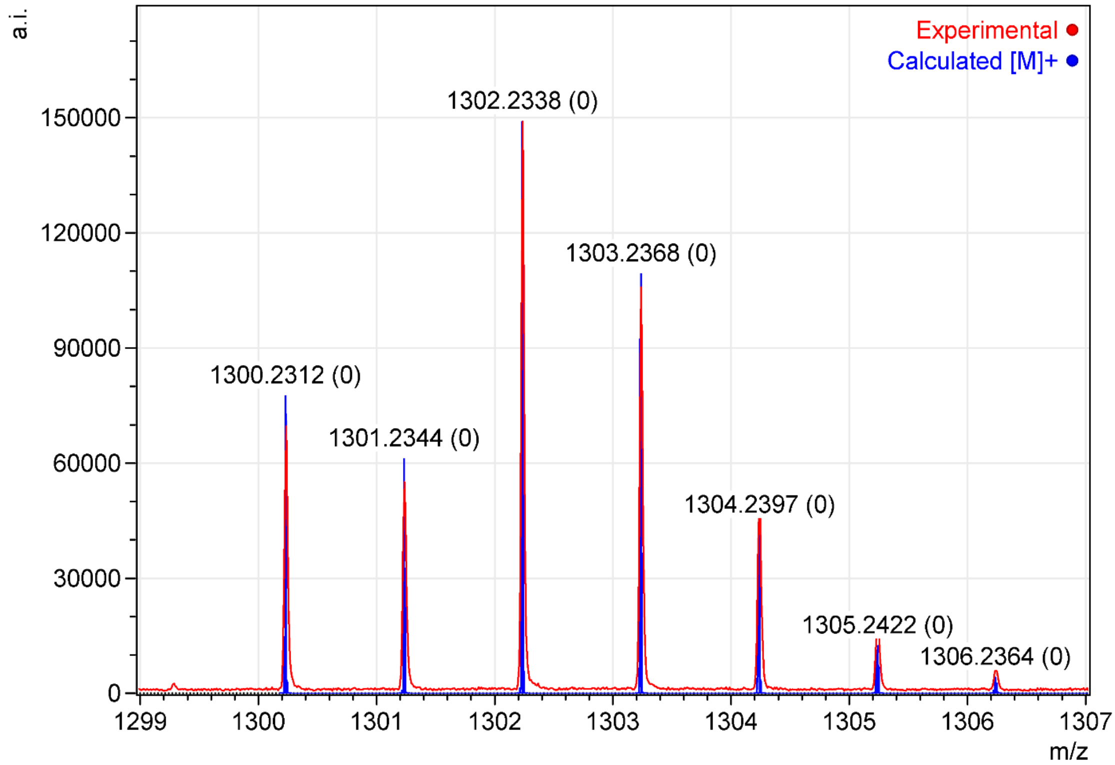


Figure S13. Fragment of experimental and simulated HR-ESI mass-spectra of complex **6**. Solution in methanol.

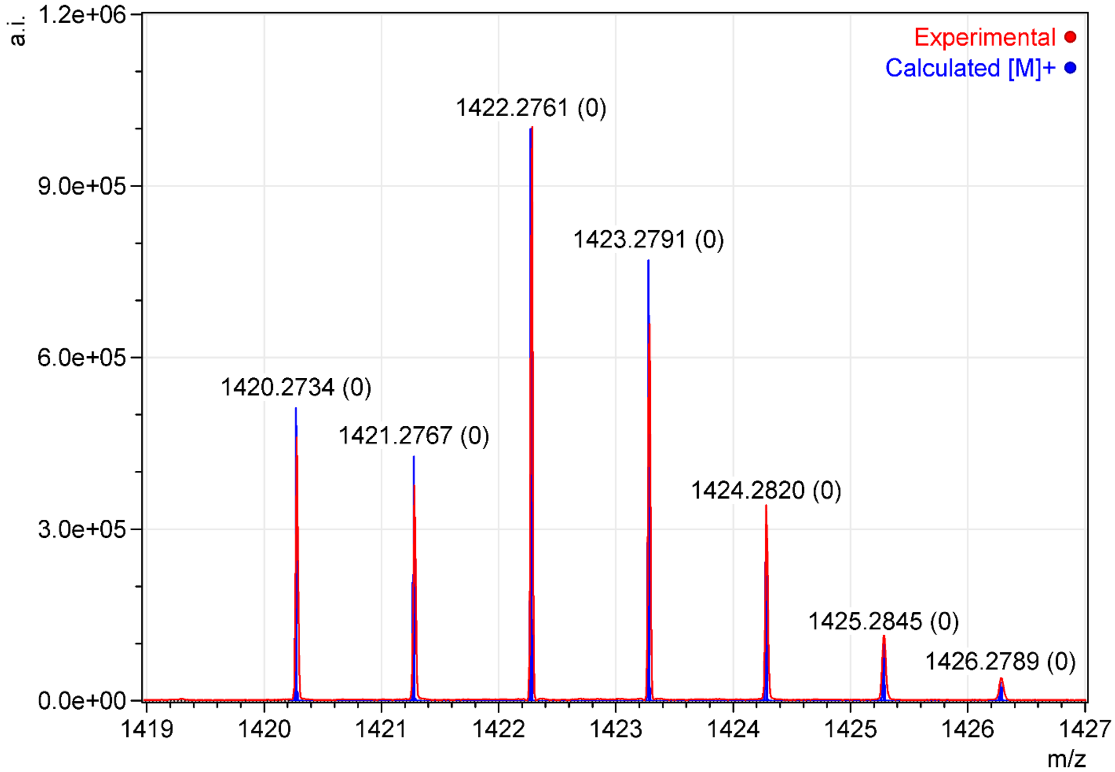


Figure S14. Fragment of experimental and simulated HR-ESI mass-spectra of complex **7**. Solution in methanol.

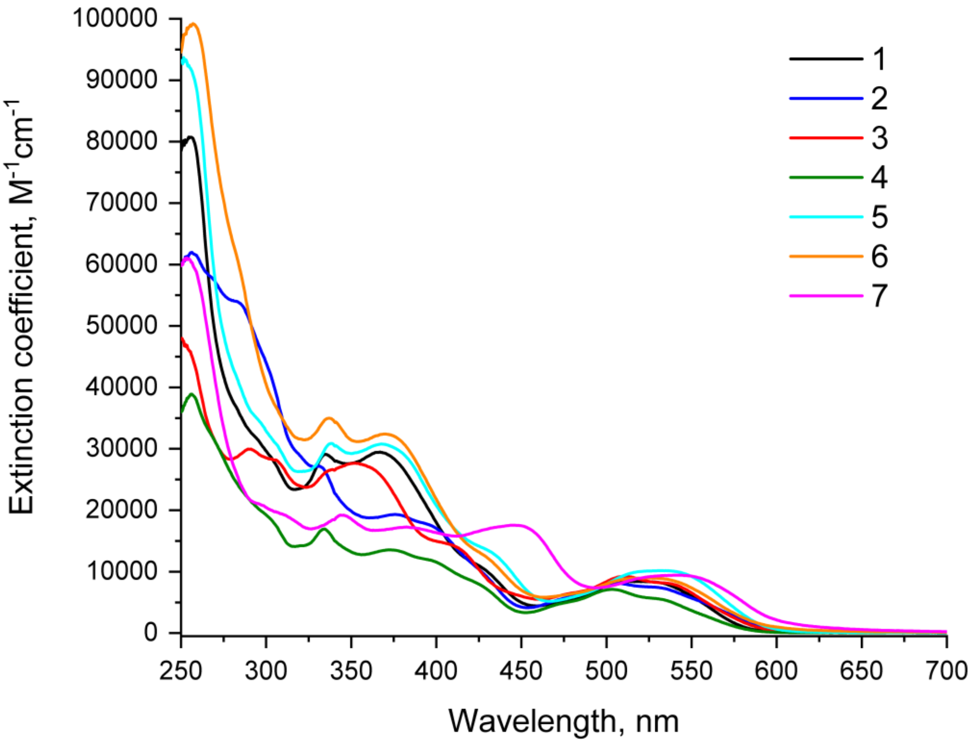


Figure S15. Absorption spectra of complexes **1-7** in aerated methanol solution at 298 K.

Table S1. Experimental and calculated data for absorption and emission wavelengths of complexes **1** and **7** in methanol solution.

| | $\lambda_{abs}$, nm ($10^{-3}$ ε) | | $\lambda_{em}$, nm | |
|---|---|---|---|---|
| | Experimental | Calculated | Exp. | Calc. |
| **1** | 255, 283sh, 335, 367, 426sh, 515 | 250, 299, 324sh, 358, 508 | 719 | 727 |
| **7** | 255, 297sh, 311sh, 338, 346, 384, 445, 539 | 255, 321sh, 342, 362sh, 433, 535 | 722 | 739 |

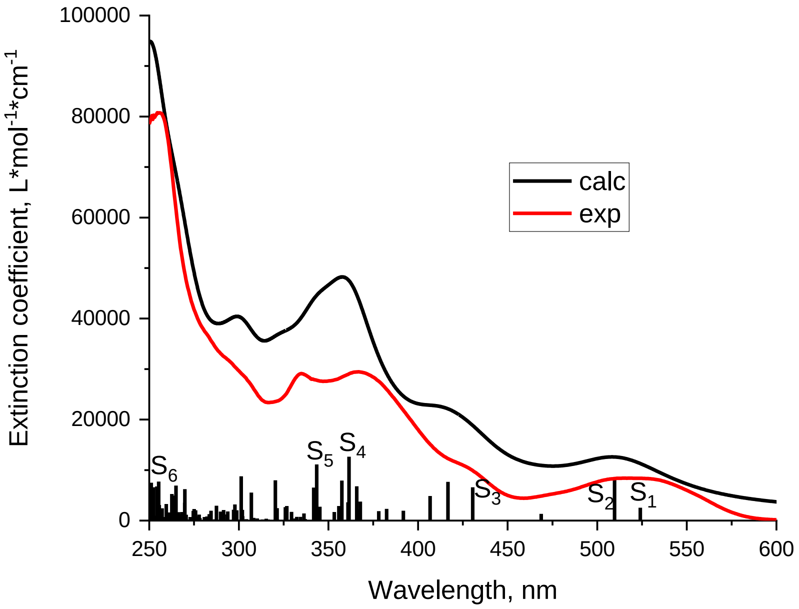


Figure S16. Absorption spectra of complex **1**: experimental (red line) and calculated (black line) with oscillator strengths of electronic transitions (bars).

Table S2. Experimental and calculated absorption maxima (λ), extinction coefficients (ε) and oscillator strengths (f) of complex **1**.

| Complex | λ, nm (exp.) | ε*$10^{-3}$, $M^{-1}cm^{-1}$ (exp.) | Transitions | λ, nm (calc.) | *f* (calc.) | Contribution of main NTO pair in transition (%) |
|---|---|---|---|---|---|---|
| **1** | 255 | 81 | $S_0 \rightarrow S_6$ | 255 | 0.10 | 23 |
| | 283sh | 45 | | | | |
| | 335 | 30 | $S_0 \rightarrow S_5$ | 343 | 0.15 | 40 |
| | 367 | 27 | $S_0 \rightarrow S_4$ | 362 | 0.17 | 63 |
| | 426sh | 12 | $S_0 \rightarrow S_3$ | 431 | 0.09 | 97 |
| | 515 | 9 | $S_0 \rightarrow S_2$ | 510 | 0.12 | 97 |
| | | | $S_0 \rightarrow S_1$ | 524 | 0.03 | 99 |

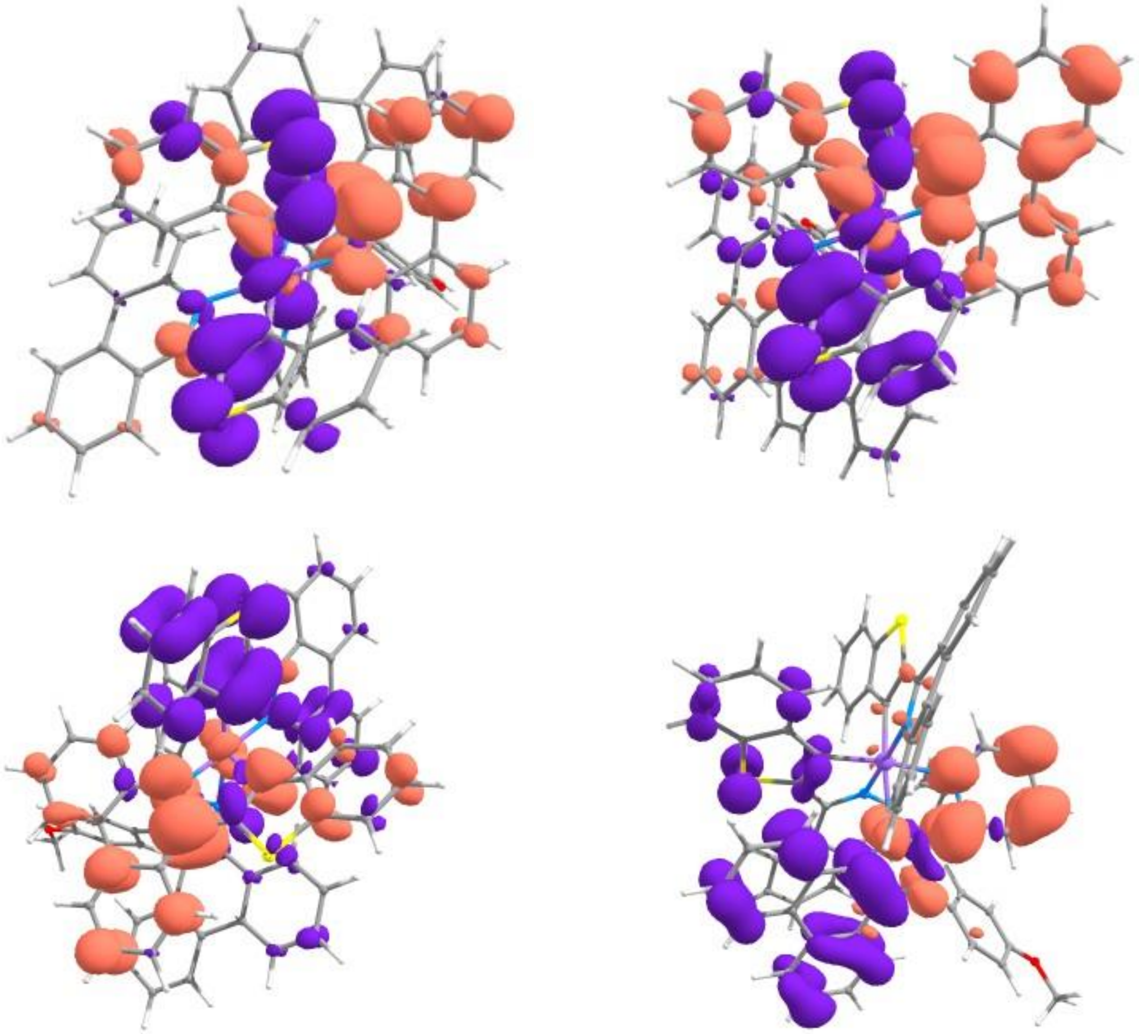

Figure S17. The depletion (violet) and increase (terracotta) of one-electron density for most intensive electronic transitions of complex **1**. Upper-left: $S_0 \rightarrow S_1$; upper-right: $S_0 \rightarrow S_2$; bottom-left: $S_0 \rightarrow S_3$; bottom-right: $S_0 \rightarrow S_4$.

Table S3. IFCT (interfragment charge transfer) of $S_0 \rightarrow S_{1\text{-}4}$ electronic transitions in complex **1**.

| $S_0 \rightarrow S_1$ transition | | | | | $S_0 \rightarrow S_2$ transition | | | | |
|---|---|---|---|---|---|---|---|---|---|
| Donor | Acceptor | | | | Donor | Acceptor | | | |
| | Ir | N^N | N^C | N^C | | Ir | N^N | N^C | N^C |
| Ir | 0.009 | 0.011 | 0.025 | 0.139 | Ir | 0.009 | 0.013 | 0.138 | 0.024 |
| N^N | 0.003 | 0.003 | 0.008 | 0.046 | N^N | 0.003 | 0.004 | 0.045 | 0.008 |
| N^C | 0.014 | 0.017 | 0.039 | 0.222 | N^C | 0.015 | 0.020 | 0.219 | 0.038 |
| N^C | 0.023 | 0.027 | 0.062 | 0.352 | N^C | 0.023 | 0.032 | 0.348 | 0.061 |
| | | | | | | | | | |
| $S_0 \rightarrow S_3$ transition | | | | | $S_0 \rightarrow S_4$ transition | | | | |
| Donor | Acceptor | | | | Donor | Acceptor | | | |
| | Ir | N^N | N^C | N^C | | Ir | N^N | N^C | N^C |
| Ir | 0.001 | 0.001 | 0.002 | 0.016 | Ir | 0.001 | 0.037 | 0.006 | 0.011 |
| N^N | 0.007 | 0.008 | 0.013 | 0.103 | N^N | 0.007 | 0.381 | 0.066 | 0.116 |
| N^C | 0.028 | 0.033 | 0.058 | 0.446 | N^C | 0.002 | 0.085 | 0.015 | 0.026 |
| N^C | 0.014 | 0.016 | 0.029 | 0.224 | N^C | 0.003 | 0.166 | 0.029 | 0.050 |

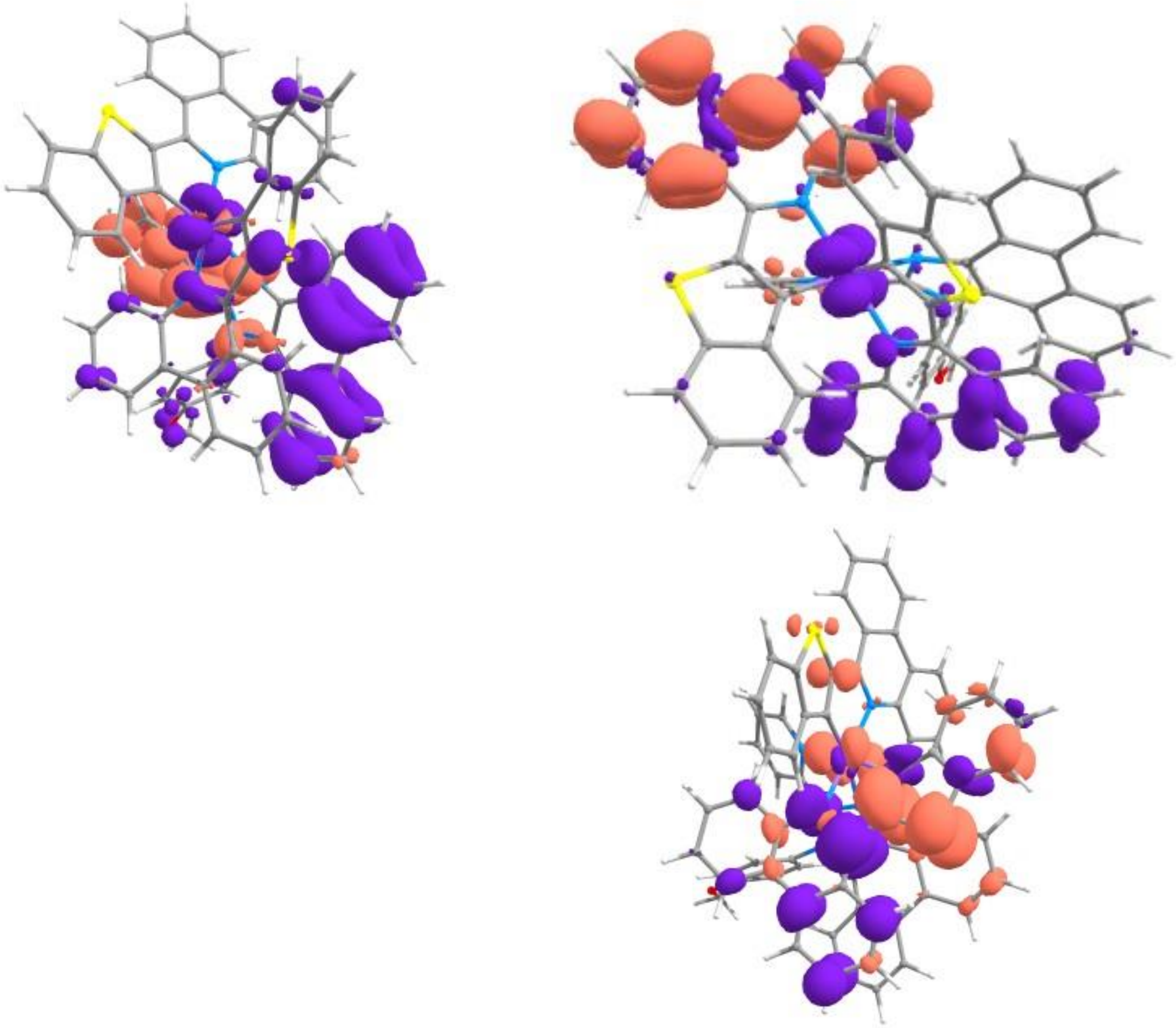

Figure S18. The depletion (violet) and increase (terracotta) of one-electron density for most intensive electronic transitions of complex **1**. Upper-left: $S_0 \rightarrow S_5$; upper-right: $S_0 \rightarrow S_6$; bottom-right: $T_1 \rightarrow S_0$.

Table S4. IFCT (interfragment charge transfer) of $S_0 \rightarrow S_{5\text{-}4}$ and $T_1 \rightarrow S_0$ electronic transitions in **1**.

| $S_0 \rightarrow S_5$ transition | | | | | $S_0 \rightarrow S_6$ transition | | | | |
|---|---|---|---|---|---|---|---|---|---|
| Donor | Acceptor | | | | Donor | Acceptor | | | |
| | Ir | N^N | N^C | N^C | | Ir | N^N | N^C | N^C |
| Ir | 0.005 | 0.104 | 0.024 | 0.044 | Ir | 0.003 | 0.030 | 0.049 | 0.027 |
| N^N | 0.009 | 0.214 | 0.050 | 0.090 | N^N | 0.003 | 0.035 | 0.056 | 0.031 |
| N^C | 0.004 | 0.082 | 0.019 | 0.034 | N^C | 0.008 | 0.080 | 0.128 | 0.072 |
| N^C | 0.008 | 0.190 | 0.044 | 0.080 | N^C | 0.013 | 0.133 | 0.213 | 0.120 |

| $T_1 \rightarrow S_0$ transition | | | | |
|---|---|---|---|---|
| Donor | Acceptor | | | |
| | Ir | N^N | N^C | N^C |
| Ir | 0.007 | 0.006 | 0.004 | 0.033 |
| N^N | 0.008 | 0.007 | 0.005 | 0.038 |
| N^C | 0.006 | 0.005 | 0.004 | 0.029 |
| N^C | 0.121 | 0.099 | 0.073 | 0.553 |

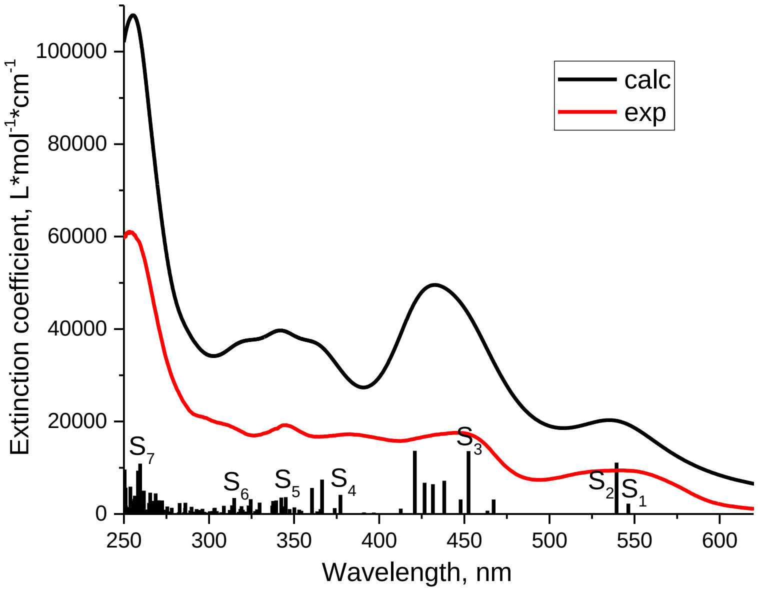


Figure S19. Absorption spectra of complex **7**: experimental (red line) and calculated (black line) with oscillator strengths of electronic transitions (bars).

Table S5. Experimental and calculated absorption maxima (λ), extinction coefficients (ε) and oscillator strengths (f) of complex **7**.

| Complex | λ, nm (exp) | $\varepsilon$*10$^{-3}$, M$^{-1}$cm$^{-1}$ (exp) | Transitions | λ, nm (calc) | *f* (calc) | Contribution of main NTO pair in transition (%) |
|---|---|---|---|---|---|---|
| **7** | 255 | 62 | $S_0 \rightarrow S_7$ | 260 | 0.19 | 30 |
| | 297sh | 18 | $S_0 \rightarrow S_6$ | 315 | 0.06 | 55 |
| | 311sh | 18 | | | | |
| | 338 | 17 | | | | |
| | 346 | 18 | $S_0 \rightarrow S_5$ | 345 | 0.06 | 59 |
| | 384 | 16 | $S_0 \rightarrow S_4$ | 377 | 0.07 | 94 |
| | 445 | 18 | $S_0 \rightarrow S_3$ | 452 | 0.24 | 92 |
| | 539 | 10 | $S_0 \rightarrow S_2$ | 539 | 0.20 | 96 |
| | | | $S_0 \rightarrow S_1$ | 546 | 0.04 | 98 |

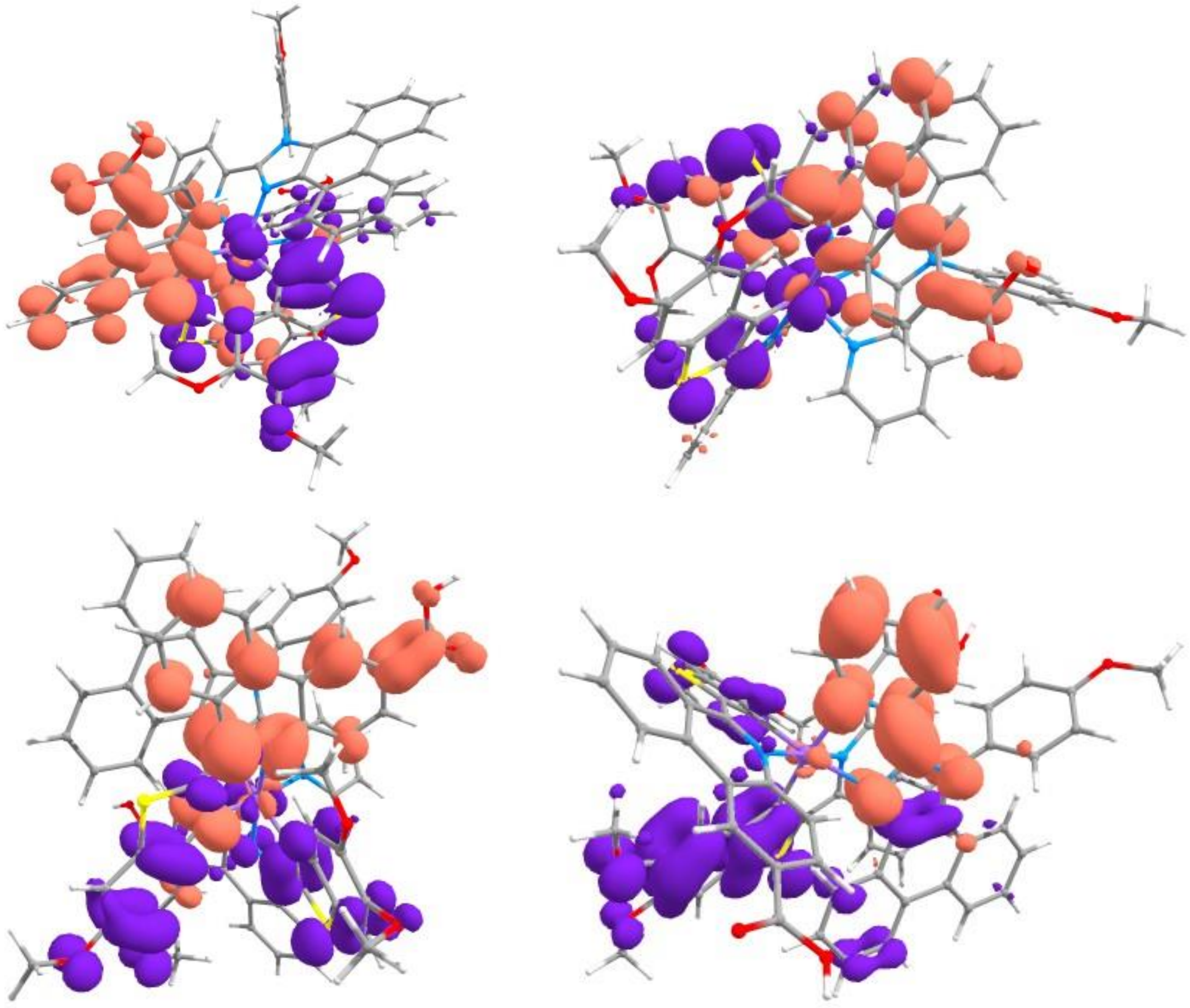

Figure S20. The depletion (violet) and increase (terracotta) of one-electron density for most intensive electronic transitions of complex **7**. Upper-left: $S_0 \rightarrow S_1$; upper-right: $S_0 \rightarrow S_2$; bottom-left: $S_0 \rightarrow S_3$; bottom-right: $S_0 \rightarrow S_4$.

Table S6. IFCT (interfragment charge transfer) of $S_0 \rightarrow S_{1\text{-}4}$ electronic transitions in complex **7**.

| $S_0 \rightarrow S_1$ transition | | | | | $S_0 \rightarrow S_2$ transition | | | | |
|---|---|---|---|---|---|---|---|---|---|
| Donor | Acceptor | | | | Donor | Acceptor | | | |
| | Ir | N^N | N^C | N^C | | Ir | N^N | N^C | N^C |
| Ir | 0.007 | 0.005 | 0.118 | 0.020 | Ir | 0.006 | 0.005 | 0.020 | 0.117 |
| N^N | 0.002 | 0.002 | 0.045 | 0.007 | N^N | 0.002 | 0.002 | 0.008 | 0.045 |
| N^C | 0.010 | 0.008 | 0.180 | 0.030 | N^C | 0.009 | 0.008 | 0.031 | 0.180 |
| N^C | 0.025 | 0.019 | 0.449 | 0.074 | N^C | 0.023 | 0.020 | 0.077 | 0.446 |
| | | | | | | | | | |
| $S_0 \rightarrow S_3$ transition | | | | | $S_0 \rightarrow S_4$ transition | | | | |
| Donor | Acceptor | | | | Donor | Acceptor | | | |
| | Ir | N^N | N^C | N^C | | Ir | N^N | N^C | N^C |
| Ir | 0.002 | 0.002 | 0.002 | 0.037 | Ir | 0.000 | 0.009 | 0.000 | 0.000 |
| N^N | 0.001 | 0.001 | 0.001 | 0.020 | N^N | 0.004 | 0.280 | 0.010 | 0.014 |
| N^C | 0.015 | 0.015 | 0.019 | 0.316 | N^C | 0.002 | 0.165 | 0.006 | 0.008 |
| N^C | 0.023 | 0.023 | 0.030 | 0.493 | N^C | 0.006 | 0.455 | 0.015 | 0.023 |

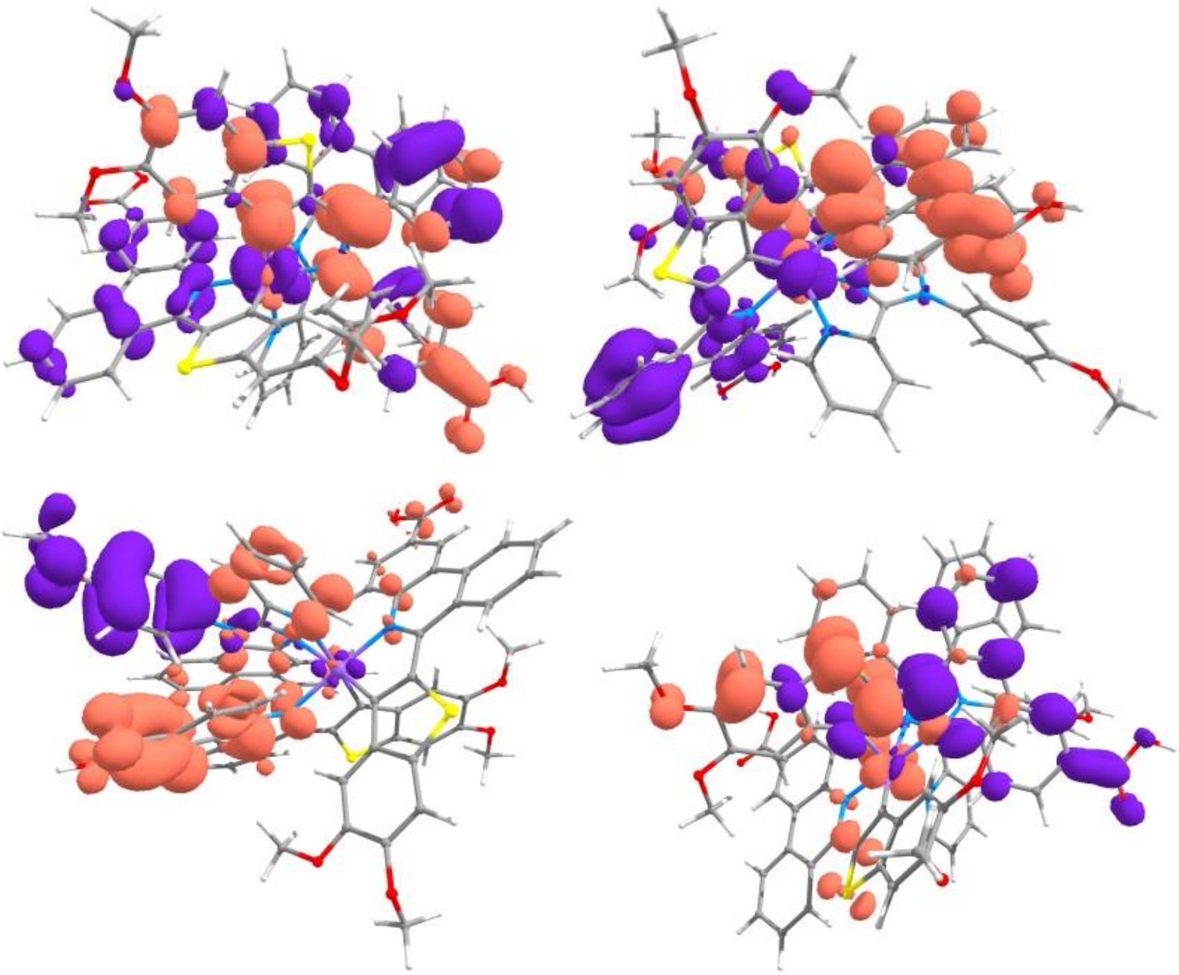

Figure S21. The depletion (violet) and increase (terracotta) of one-electron density for most intensive electronic transitions of complex **7**. Upper-left: $S_0 \rightarrow S_5$; upper-right: $S_0 \rightarrow S_6$; bottom-left: $S_0 \rightarrow S_7$, bottom-right: $T_1 \rightarrow S_0$.

Table S6. IFCT (interfragment charge transfer) of $S_0 \rightarrow S_{5\text{-}4}$ and $T_1 \rightarrow S_0$ electronic transitions in **7**.

| $S_0 \rightarrow S_5$ transition | | | | | $S_0 \rightarrow S_6$ transition | | | | |
|---|---|---|---|---|---|---|---|---|---|
| Donor | Acceptor | | | | Donor | Acceptor | | | |
| | Ir | N^N | N^C | N^C | | Ir | N^N | N^C | N^C |
| Ir | 0.005 | 0.028 | 0.008 | 0.084 | Ir | 0.005 | 0.028 | 0.008 | 0.084 |
| N^N | 0.004 | 0.022 | 0.006 | 0.064 | N^N | 0.004 | 0.022 | 0.006 | 0.064 |
| N^C | 0.010 | 0.054 | 0.016 | 0.159 | N^C | 0.010 | 0.054 | 0.016 | 0.159 |
| N^C | 0.022 | 0.122 | 0.035 | 0.362 | N^C | 0.022 | 0.122 | 0.035 | 0.362 |
| | | | | | | | | | |
| $S_0 \rightarrow S_7$ transition | | | | | $T_1 \rightarrow S_0$ transition | | | | |
| Donor | Acceptor | | | | Donor | Acceptor | | | |
| | Ir | N^N | N^C | N^C | | Ir | N^N | N^C | N^C |
| Ir | 0.001 | 0.012 | 0.013 | 0.020 | Ir | 0.005 | 0.003 | 0.005 | 0.030 |
| N^N | 0.014 | 0.130 | 0.144 | 0.229 | N^N | 0.005 | 0.004 | 0.005 | 0.032 |
| N^C | 0.007 | 0.063 | 0.069 | 0.110 | N^C | 0.005 | 0.004 | 0.005 | 0.033 |
| N^C | 0.005 | 0.047 | 0.052 | 0.084 | N^C | 0.099 | 0.069 | 0.096 | 0.600 |

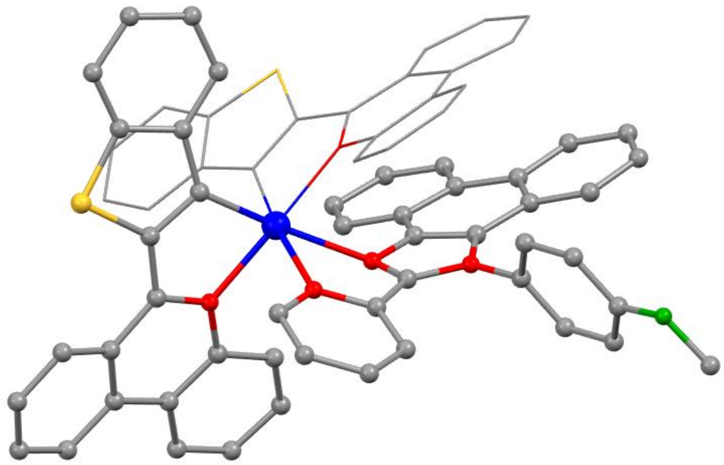

Figure S22. Structures of complexe **1**. Colors of atoms: Ir – blue, S – yellow, O – green, N – red, C –grey. Hydrogen atoms are omitted for clarity, one of the N^C ligands is shown in wireframe style to simplify visualization.

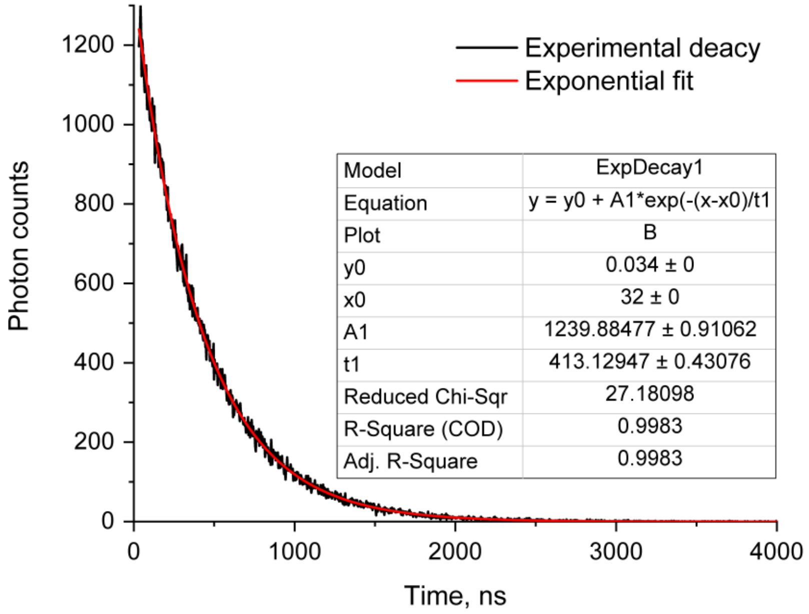


Figure S23. Phosphorescence intensity decay curve and its monoexponential fit for complex **1** (“t1” lifetime in ns). Aerated solution in methanol, temperature 298 K, concentration of complex 10 uM, excitation at 355 nm, acquisition at 720 nm.

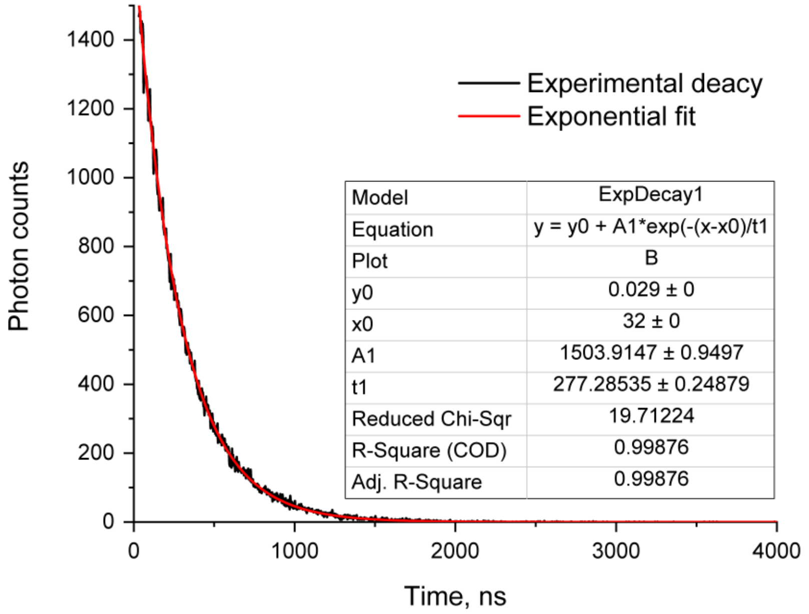


Figure S24. Phosphorescence intensity decay curve and its monoexponential fit for complex **2** (“t1” lifetime in ns). Aerated solution in methanol, temperature 298 K, concentration of complex 10 uM, excitation at 355 nm, acquisition at 720 nm.

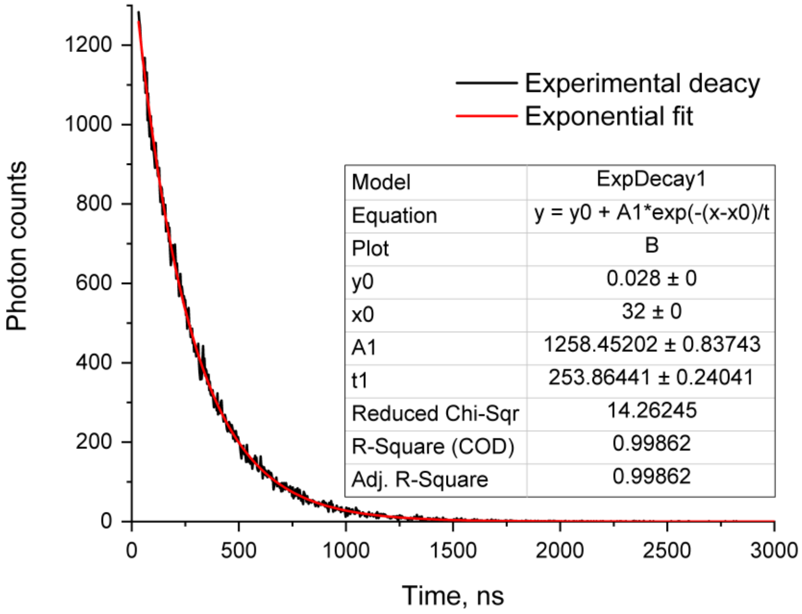


Figure S25. Phosphorescence intensity decay curve and its monoexponential fit for complex **3** (“t1” lifetime in ns). Aerated solution in methanol, temperature 298 K, concentration of complex 10 uM, excitation at 355 nm, acquisition at 720 nm.

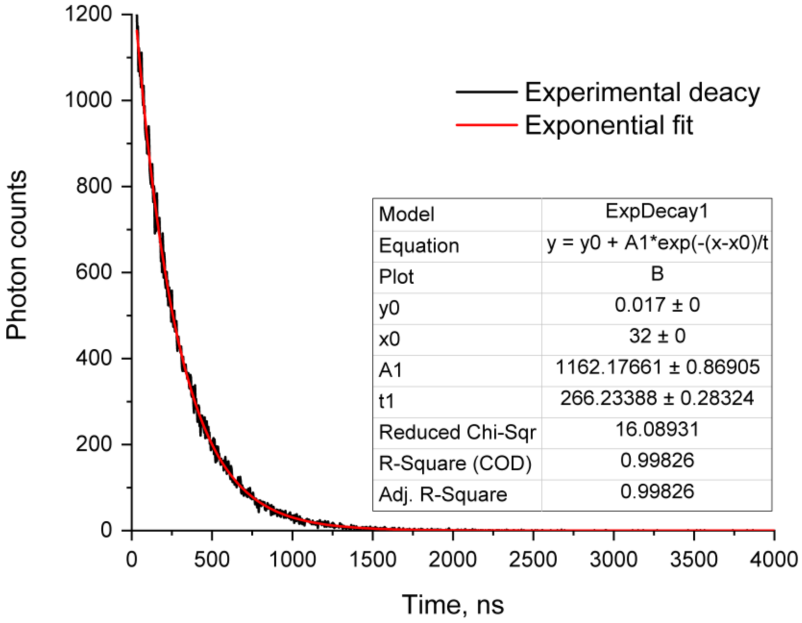


Figure S26. Phosphorescence intensity decay curve and its monoexponential fit for complex **4** (“t1” lifetime in ns). Aerated solution in methanol, temperature 298 K, concentration of complex 10 uM, excitation at 355 nm, acquisition at 720 nm.

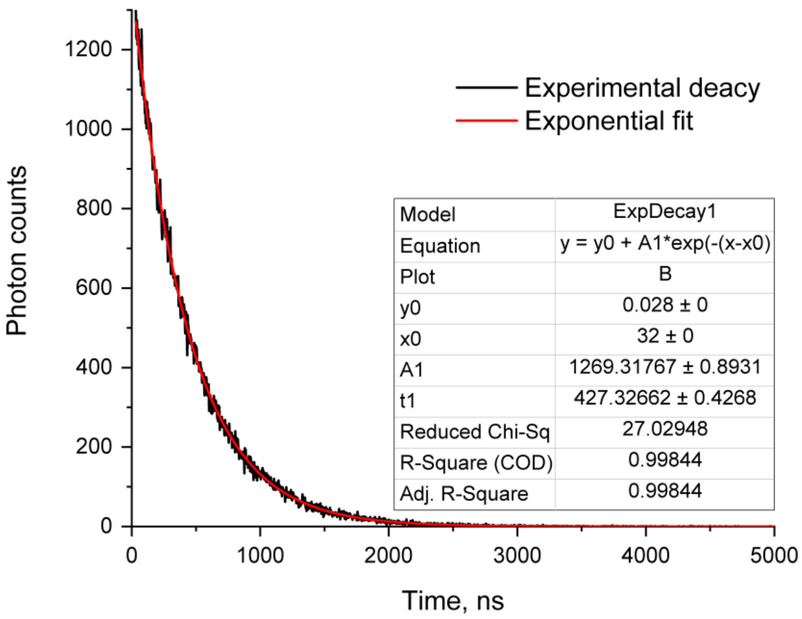


Figure S27. Phosphorescence intensity decay curve and its monoexponential fit for complex **5** ("t1" lifetime in ns). Aerated solution in methanol, temperature 298 K, concentration of complex 10 uM, excitation at 355 nm, acquisition at 720 nm.

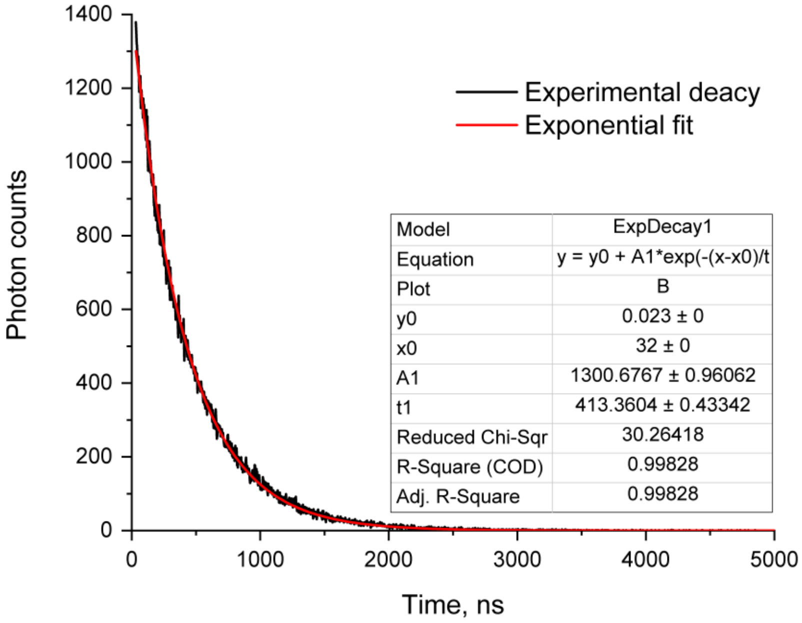


Figure S28. Phosphorescence intensity decay curve and its monoexponential fit for complex **6** ("t1" lifetime in ns). Aerated solution in methanol, temperature 298 K, concentration of complex 10 uM, excitation at 355 nm, acquisition at 720 nm.

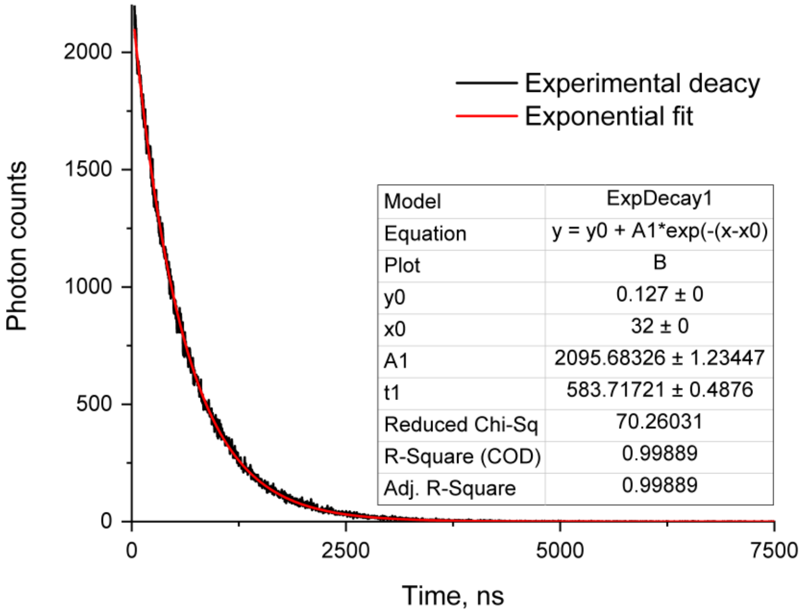


Figure S29. Phosphorescence intensity decay curve and its monoexponential fit for complex **7** ("t1" lifetime in ns). Aerated solution in methanol, temperature 298 K, concentration of complex 10 uM, excitation at 355 nm, acquisition at 720 nm.

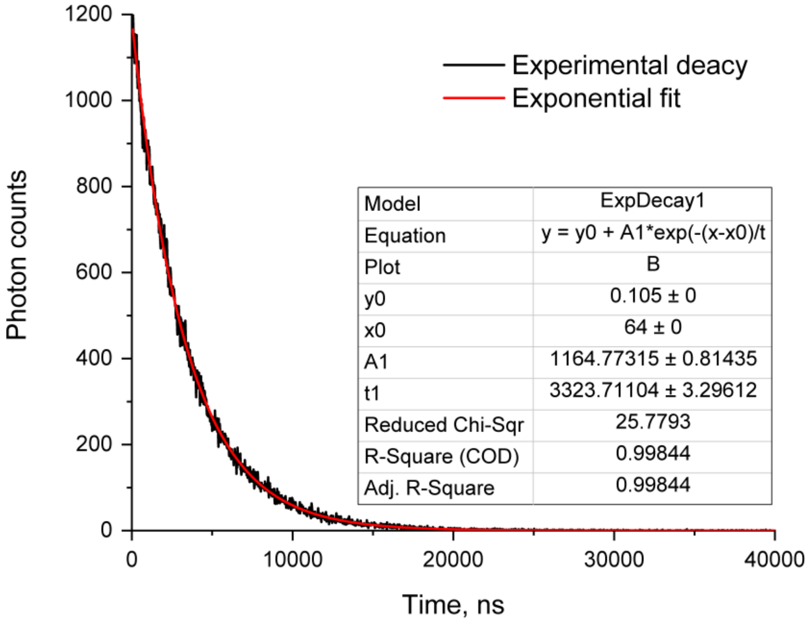


Figure S30. Phosphorescence intensity decay curve and its monoexponential fit for complex **1** ("t1" lifetime in ns). Argon saturated solution in methanol, temperature 298 K, concentration of complex 10 uM, excitation at 355 nm, acquisition at 720 nm.

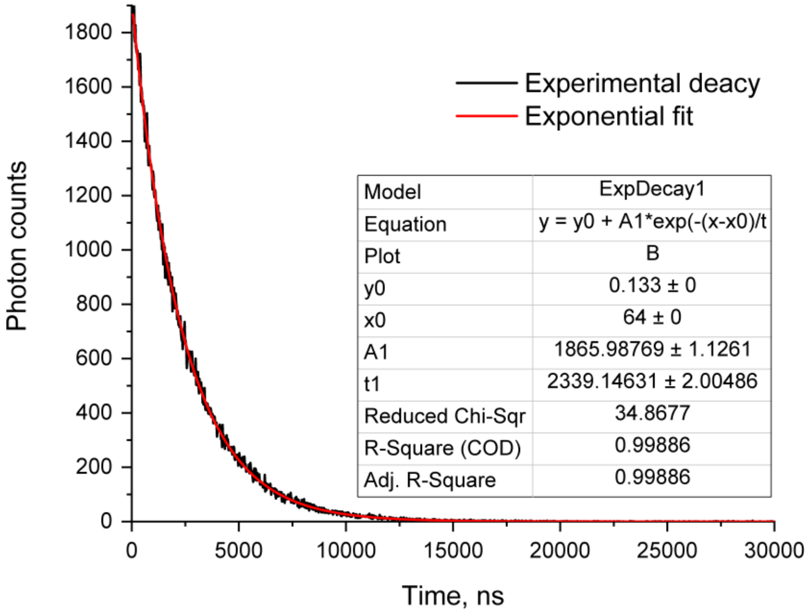


Figure S31. Phosphorescence intensity decay curve and its monoexponential fit for complex **2** (“t1” lifetime in ns). Argon saturated solution in methanol, temperature 298 K, concentration of complex 10 uM, excitation at 355 nm, acquisition at 720 nm.

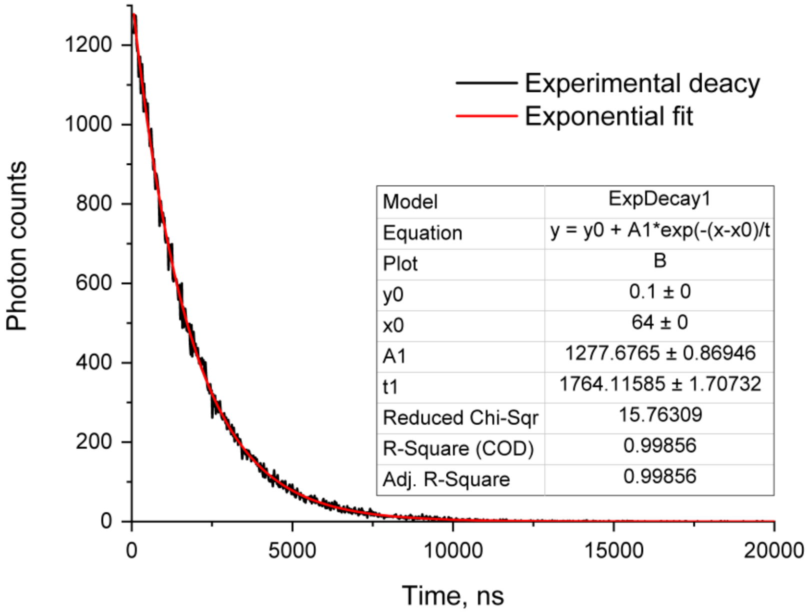


Figure S32. Phosphorescence intensity decay curve and its monoexponential fit for complex **3** (“t1” lifetime in ns). Argon saturated solution in methanol, temperature 298 K, concentration of complex 10 uM, excitation at 355 nm, acquisition at 720 nm.

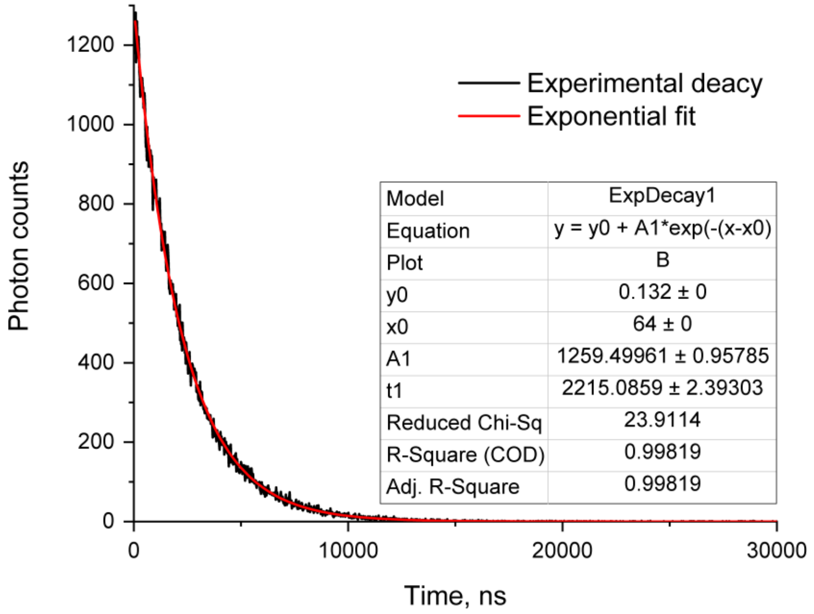


Figure S33. Phosphorescence intensity decay curve and its monoexponential fit for complex **4** (“t1” lifetime in ns). Argon saturated solution in methanol, temperature 298 K, concentration of complex 10 uM, excitation at 355 nm, acquisition at 720 nm.

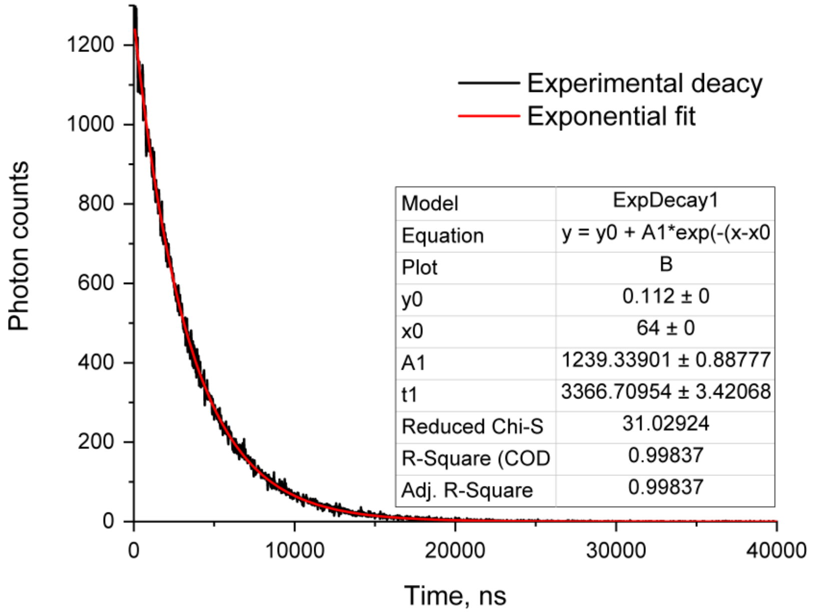


Figure S34. Phosphorescence intensity decay curve and its monoexponential fit for complex **5** (“t1” lifetime in ns). Argon saturated solution in methanol, temperature 298 K, concentration of complex 10 uM, excitation at 355 nm, acquisition at 720 nm.

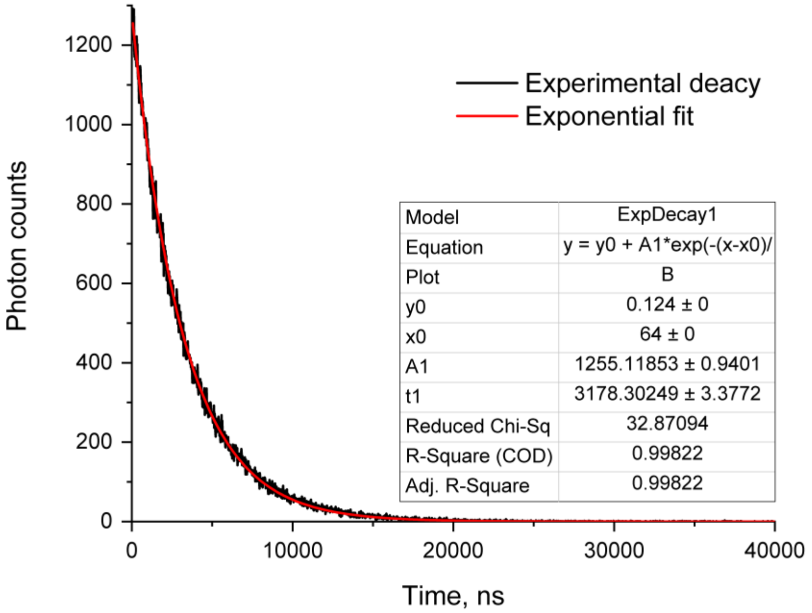


Figure S35. Phosphorescence intensity decay curve and its monoexponential fit for complex **6** (“t1” lifetime in ns). Argon saturated solution in methanol, temperature 298 K, concentration of complex 10 uM, excitation at 355 nm, acquisition at 720 nm.

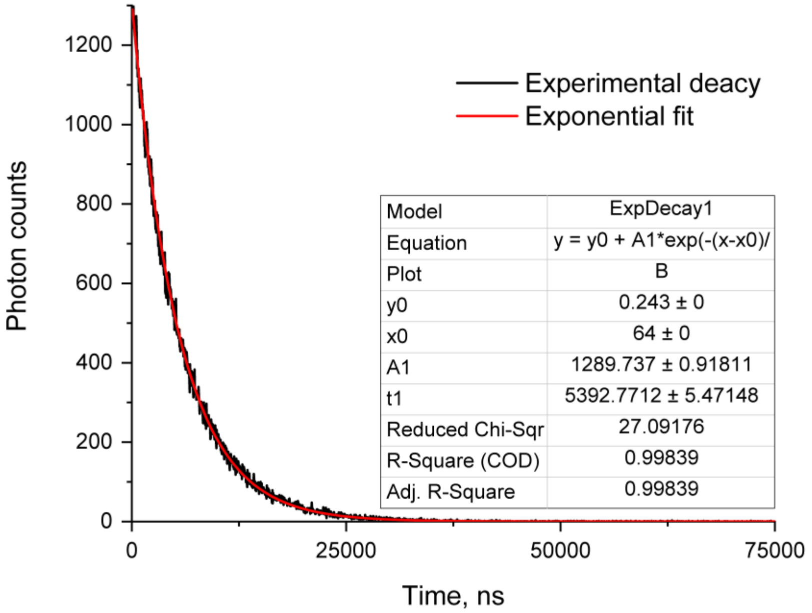


Figure S36. Phosphorescence intensity decay curve and its monoexponential fit for complex **7** (“t1” lifetime in ns). Argon saturated solution in methanol, temperature 298 K, concentration of complex 10 uM, excitation at 355 nm, acquisition at 720 nm.

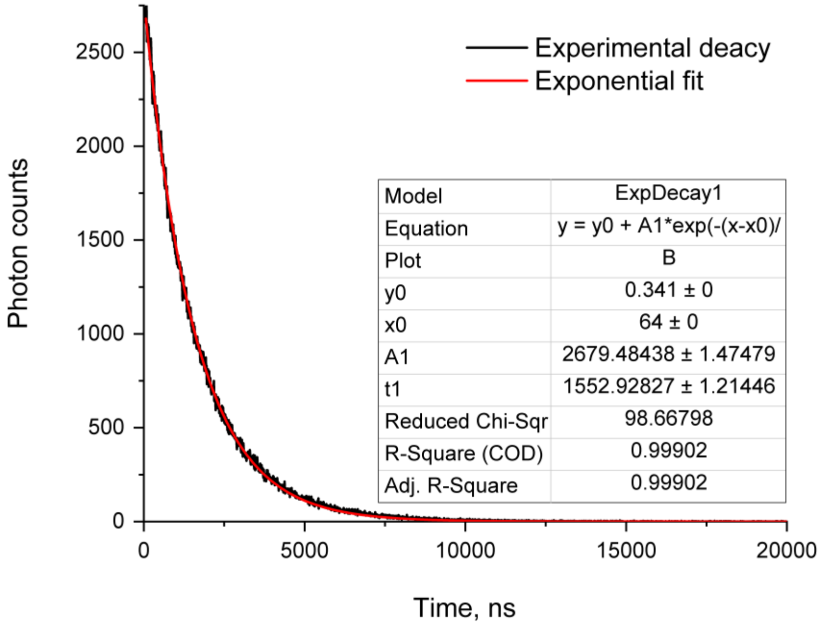


Figure S37. Phosphorescence intensity decay curve and its monoexponential fit for complex **1** ("t1" lifetime in ns). Aqueous DMEM-FBS-PEG(200) solution. Partial pressure of oxygen 152 mmHg, temperature 310 K, excitation at 355 nm, acquisition at 720 nm. Content of DMEM 88.5 % v/v, FBS – 10% v/v, PEG(200) – 1.5% v/v, concentration of **1** – 5 μM.

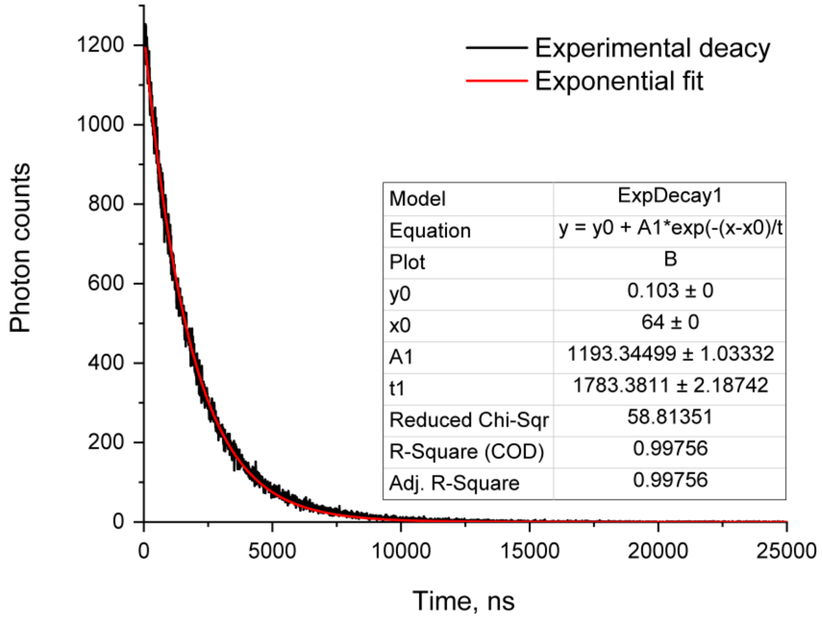


Figure S38. Phosphorescence intensity decay curve and its monoexponential fit for complex **1** ("t1" lifetime in ns). Aqueous DMEM-FBS-PEG(200) solution. Partial pressure of oxygen 107 mmHg, temperature 310 K, excitation at 355 nm, acquisition at 720 nm. Content of DMEM 88.5 % v/v, FBS – 10% v/v, PEG(200) – 1.5% v/v, concentration of **1** – 5 μM.

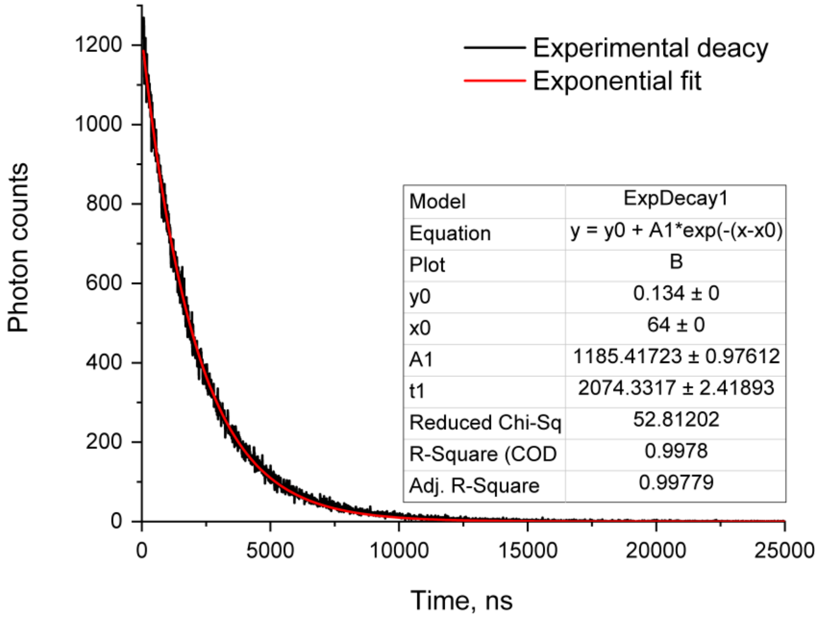


Figure S39. Phosphorescence intensity decay curve and its monoexponential fit for complex **1** (“t1” lifetime in ns). Aqueous DMEM-FBS-PEG(200) solution. Partial pressure of oxygen 54.8 mmHg, temperature 310 K, excitation at 355 nm, acquisition at 720 nm. Content of DMEM 88.5 % v/v, FBS – 10% v/v, PEG(200) – 1.5% v/v, concentration of **1** – 5 μM.

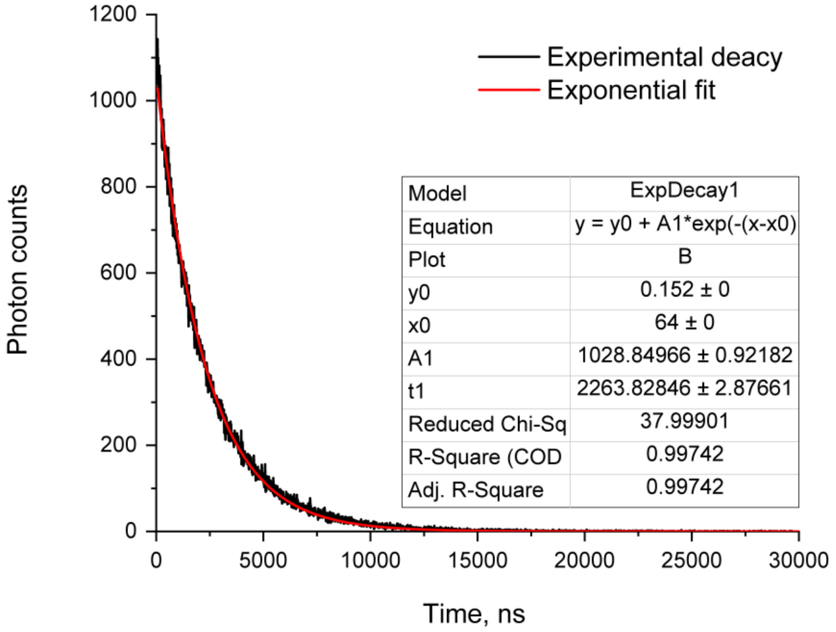


Figure S40. Phosphorescence intensity decay curve and its monoexponential fit for complex **1** (“t1” lifetime in ns). Aqueous DMEM-FBS-PEG(200) solution. Partial pressure of oxygen 31.1 mmHg, temperature 310 K, excitation at 355 nm, acquisition at 720 nm. Content of DMEM 88.5 % v/v, FBS – 10% v/v, PEG(200) – 1.5% v/v, concentration of **1** – 5 μM.

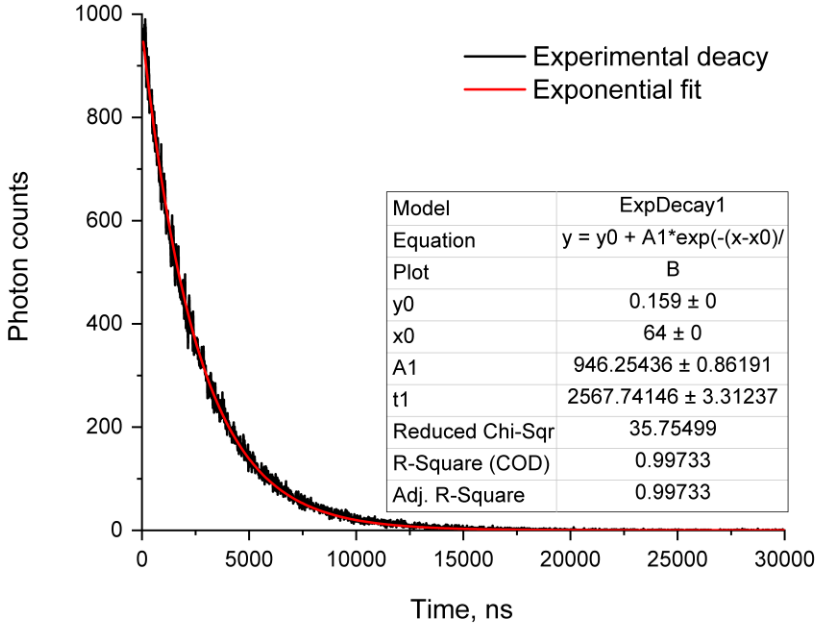


Figure S41. Phosphorescence intensity decay curve and its monoexponential fit for complex **1** ("t1" lifetime in ns). Aqueous DMEM-FBS-PEG(200) solution. Partial pressure of oxygen 0.39 mmHg, temperature 310 K, excitation at 355 nm, acquisition at 720 nm. Content of DMEM 88.5 % v/v, FBS – 10% v/v, PEG(200) – 1.5% v/v, concentration of **1** – 5 µM.

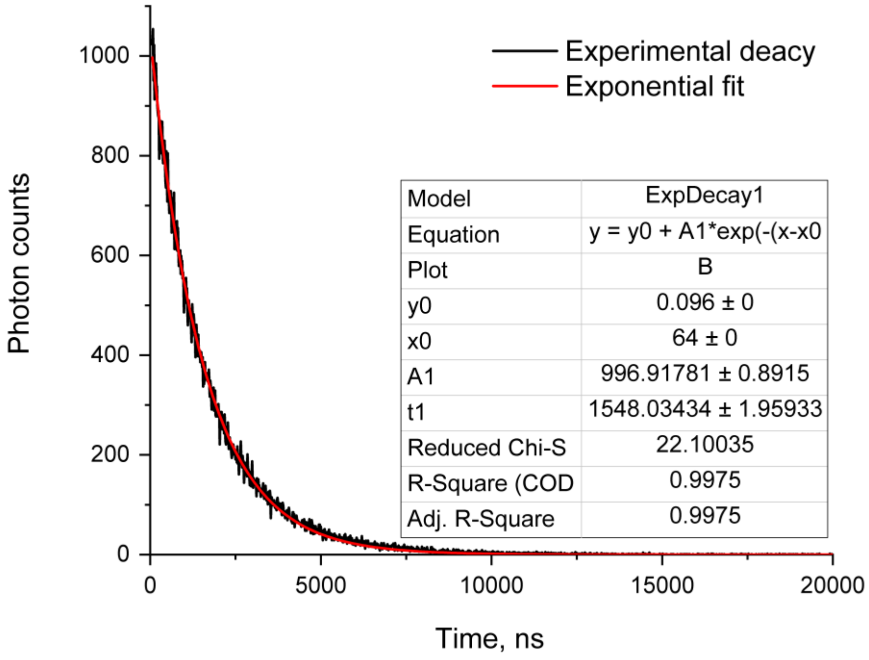


Figure S42. Phosphorescence intensity decay curve and its monoexponential fit for complex **1** ("t1" lifetime in ns). Aqueous PBS-BSA-PEG(200) solution. Partial pressure of oxygen 150 mmHg, temperature 310 K, excitation at 355 nm, acquisition at 720 nm. Content of PBS (0.01 M pH 7.4) 98.5 % v/v, BSA 50 µM, PEG(200) – 1.5% v/v, concentration of **1** – 5 µM.

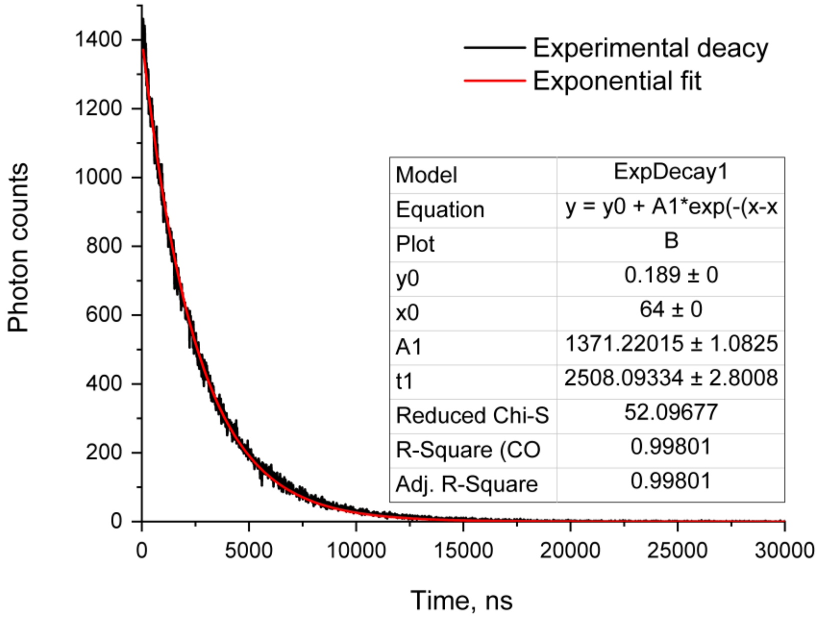


Figure S43. Phosphorescence intensity decay curve and its monoexponential fit for complex **1** (“t1” lifetime in ns). Aqueous PBS-BSA-PEG(200) solution. Partial pressure of oxygen 0.40 mmHg, temperature 310 K, excitation at 355 nm, acquisition at 720 nm. Content of PBS (0.01 M pH 7.4) 98.5 % v/v, BSA 50 µM, PEG(200) – 1.5% v/v, concentration of **1** – 5 µM.

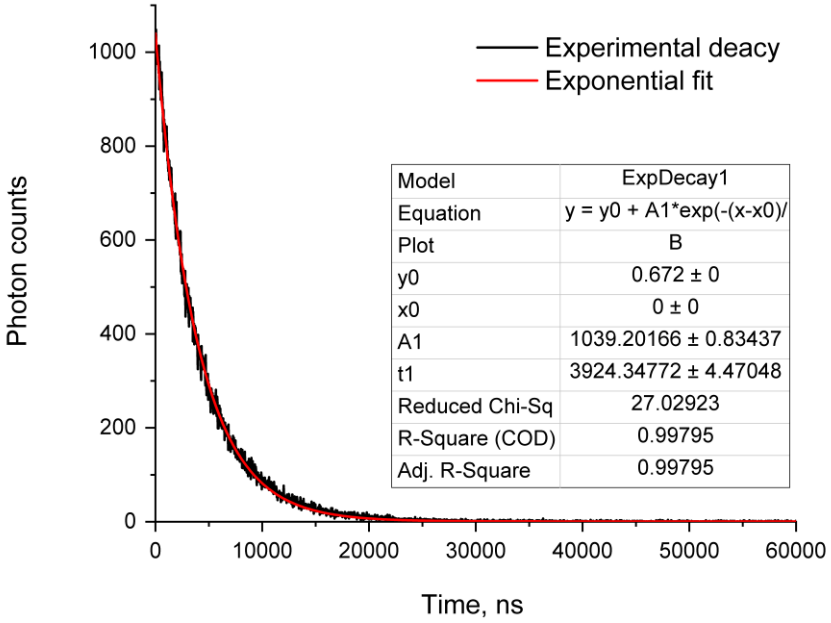


Figure S44. Phosphorescence intensity decay curve and its monoexponential fit for complex **7** (“t1” lifetime in ns). Aqueous DMEM-FBS-PEG(200) solution. Partial pressure of oxygen 145 mmHg, temperature 310 K, excitation at 355 nm, acquisition at 720 nm. Content of DMEM 87.5 % v/v, FBS – 10% v/v, PEG(200) – 2.5% v/v, concentration of **7** – 10 µM.

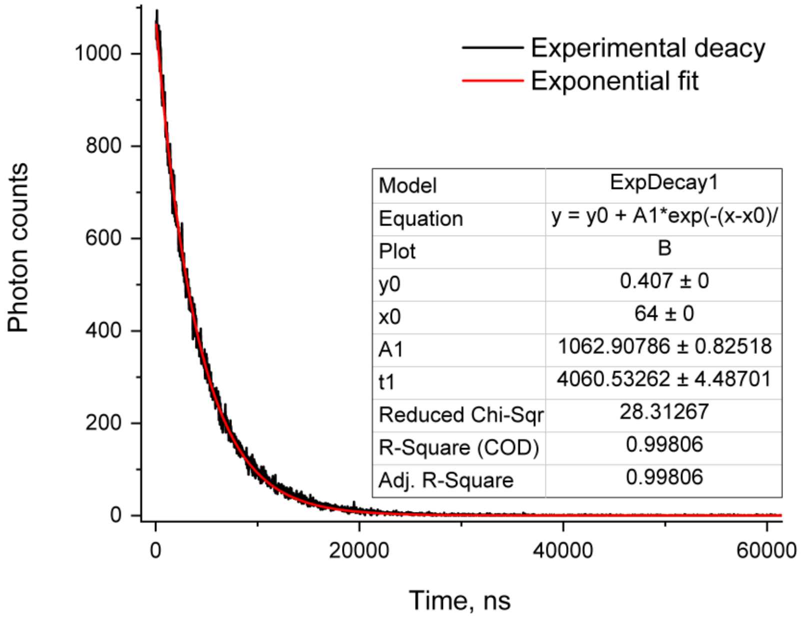


Figure S45. Phosphorescence intensity decay curve and its monoexponential fit for complex **7** (“t1” lifetime in ns). Aqueous DMEM-FBS-PEG(200) solution. Partial pressure of oxygen 128 mmHg, temperature 310 K, excitation at 355 nm, acquisition at 720 nm. Content of DMEM 87.5 % v/v, FBS – 10% v/v, PEG(200) – 2.5% v/v, concentration of **7** – 10 µM.

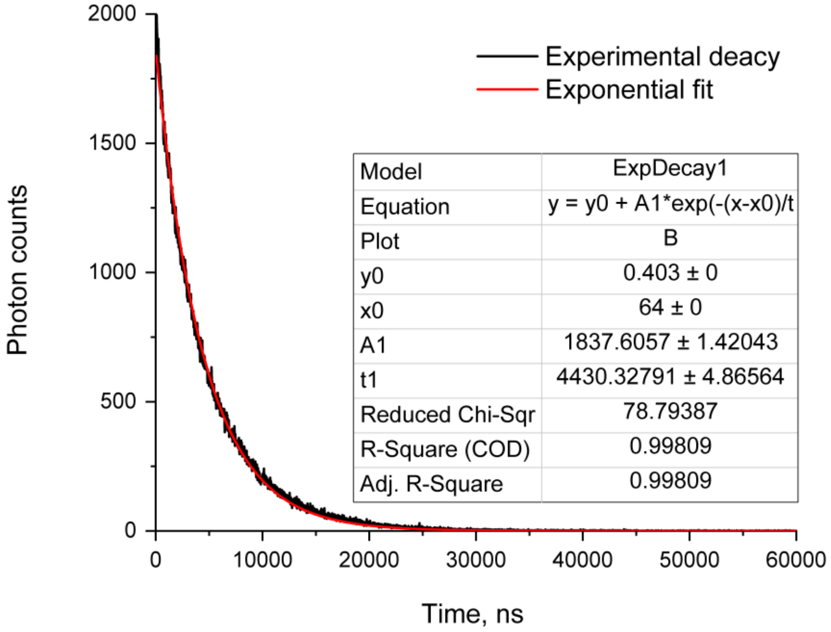


Figure S46. Phosphorescence intensity decay curve and its monoexponential fit for complex **7** (“t1” lifetime in ns). Aqueous DMEM-FBS-PEG(200) solution. Partial pressure of oxygen 82.9 mmHg, temperature 310 K, excitation at 355 nm, acquisition at 720 nm. Content of DMEM 87.5 % v/v, FBS – 10% v/v, PEG(200) – 2.5% v/v, concentration of **7** – 10 µM.

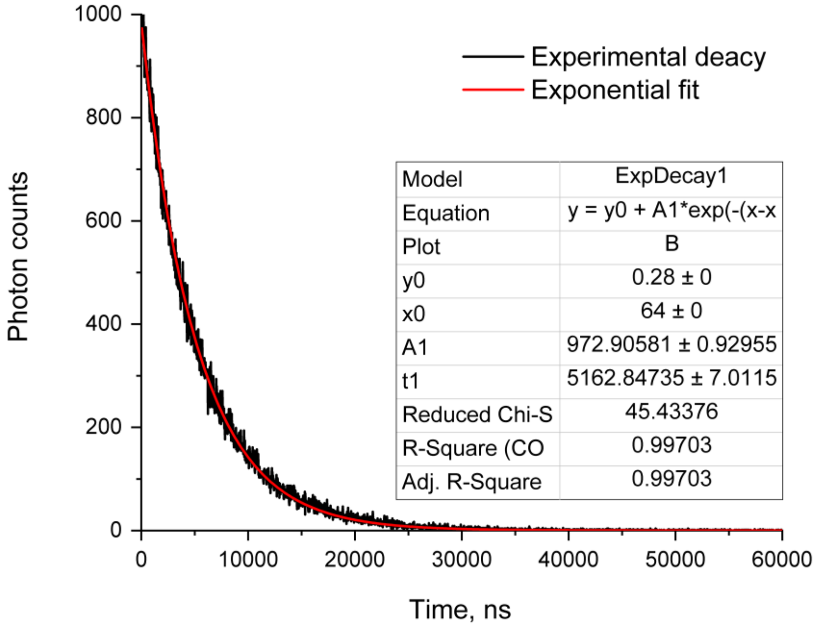


Figure S47. Phosphorescence intensity decay curve and its monoexponential fit for complex **7** (“t1” lifetime in ns). Aqueous DMEM-FBS-PEG(200) solution. Partial pressure of oxygen 21.1 mmHg, temperature 310 K, excitation at 355 nm, acquisition at 720 nm. Content of DMEM 87.5 % v/v, FBS – 10% v/v, PEG(200) – 2.5% v/v, concentration of **7** – 10 μM.

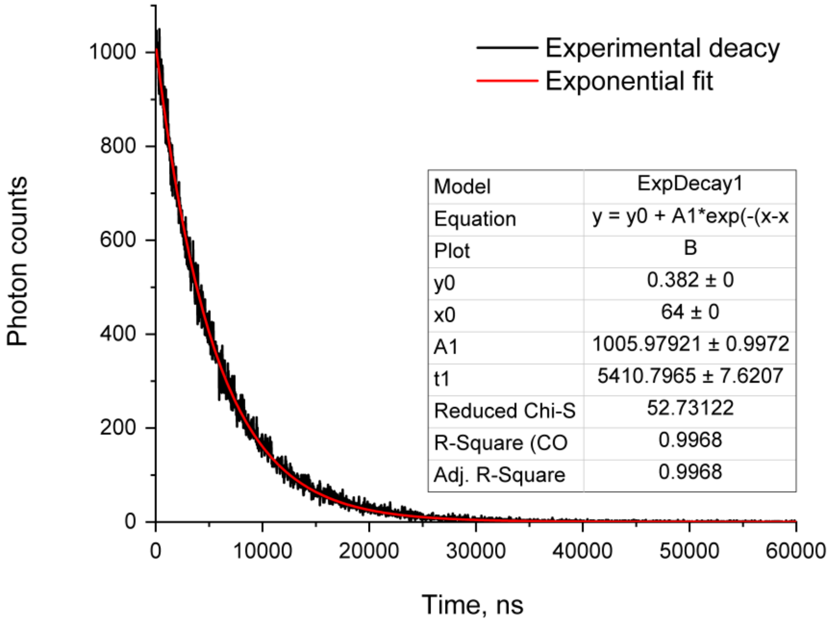


Figure S48. Phosphorescence intensity decay curve and its monoexponential fit for complex **7** (“t1” lifetime in ns). Aqueous DMEM-FBS-PEG(200) solution. Partial pressure of oxygen 0.36 mmHg, temperature 310 K, excitation at 355 nm, acquisition at 720 nm. Content of DMEM 87.5 % v/v, FBS – 10% v/v, PEG(200) – 2.5% v/v, concentration of **7** – 10 μM.

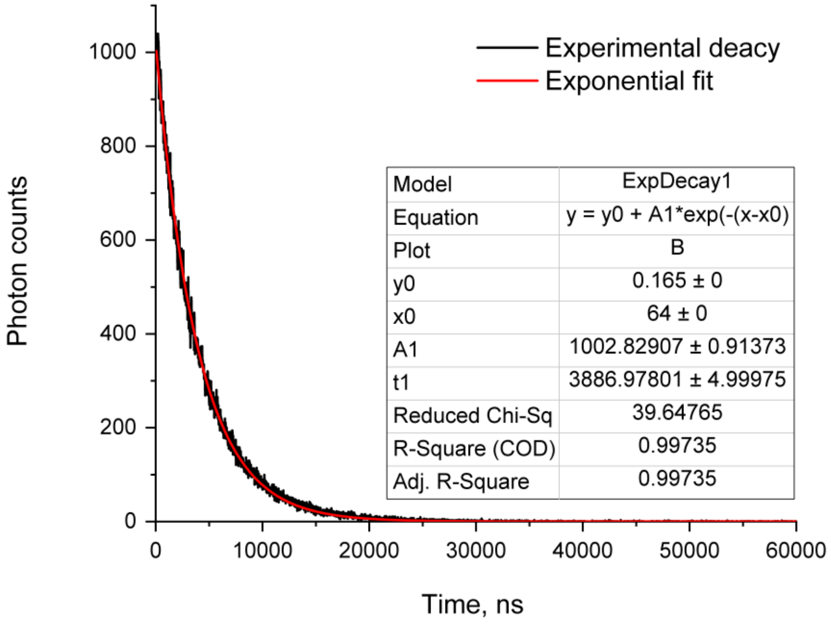


Figure S49. Phosphorescence intensity decay curve and its monoexponential fit for complex **7** (“t1” lifetime in ns). Aqueous PBS-BSA-PEG(200) solution. Partial pressure of oxygen 149 mmHg, temperature 310 K, excitation at 355 nm, acquisition at 720 nm. Content of PBS (0.01 M pH 7.4) 97.5 % v/v, BSA 50 μM, PEG(200) – 2.5% v/v, concentration of **7** – 10 μM.

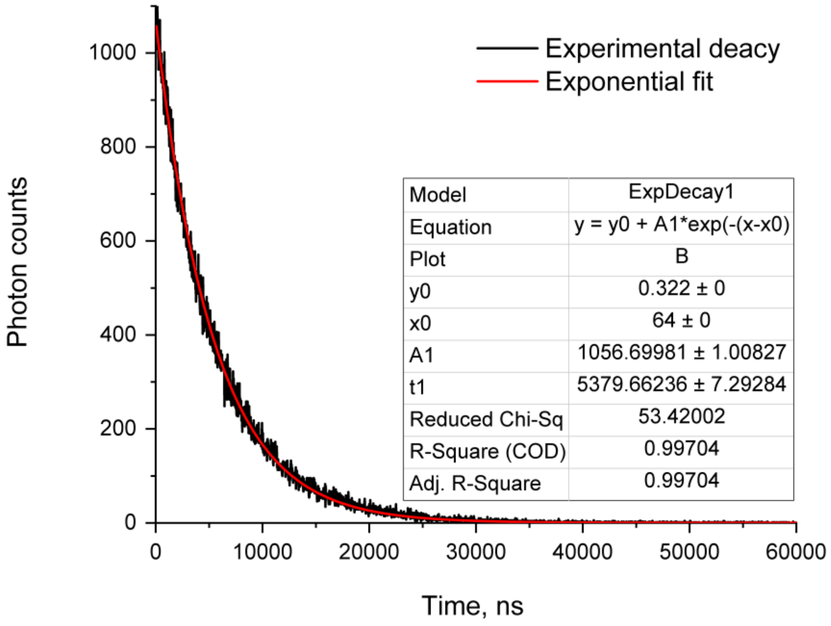


Figure S50. Phosphorescence intensity decay curve and its monoexponential fit for complex **7** (“t1” lifetime in ns). Aqueous PBS-BSA-PEG(200) solution. Partial pressure of oxygen 0.38 mmHg, temperature 310 K, excitation at 355 nm, acquisition at 720 nm. Content of PBS (0.01 M pH 7.4) 97.5 % v/v, BSA 50 μM, PEG(200) – 2.5% v/v, concentration of **7** – 10 μM.

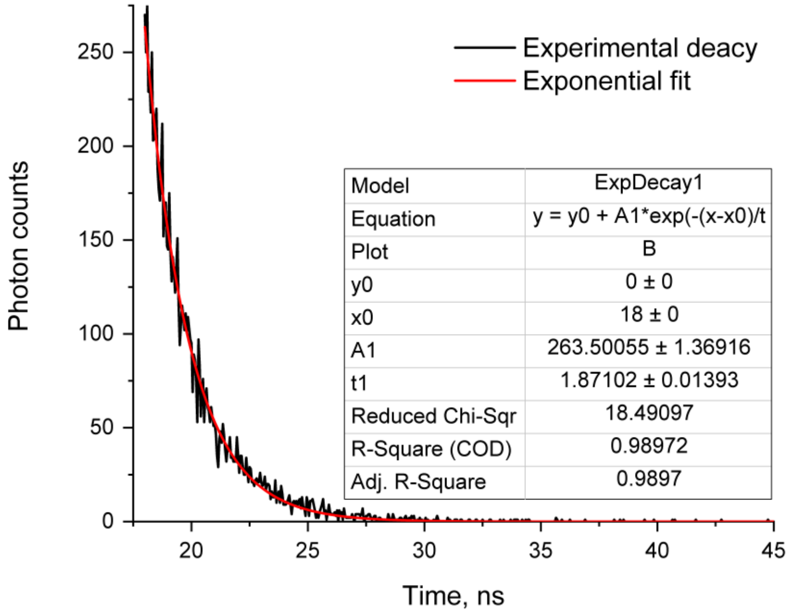


Figure S51. Example of phosphorescence intensity decay curve and its monoexponential fit for complex **1** (“t1” lifetime in µs) in PLIM (selected pixels). CHO-K1 cells incubated with DMEM-FBS-PEG(200) solution of complex **1** for 24 h. Normoxia, temperature 310 K, excitation at 405 nm. Content of DMEM 88.5 % v/v, FBS – 10% v/v, PEG(200) – 1.5% v/v, **1** – 5 µM.

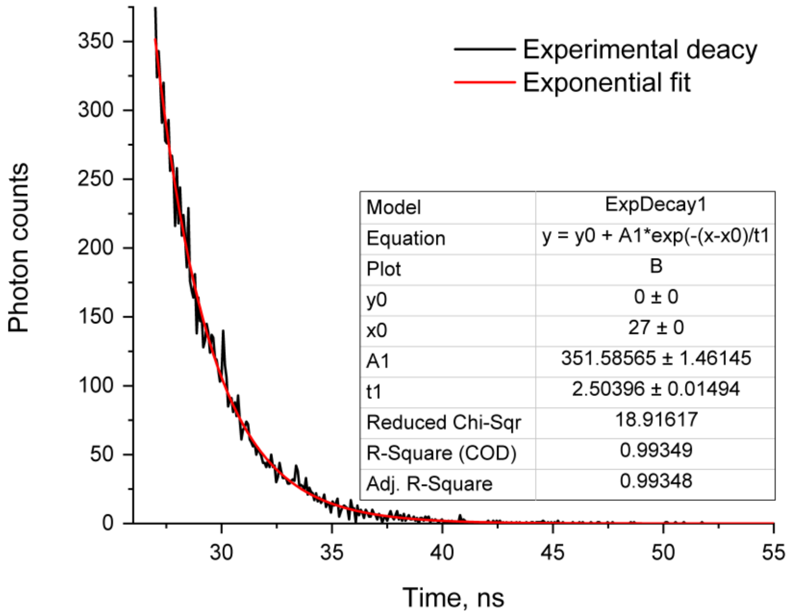


Figure S52. Example of phosphorescence intensity decay curve and its monoexponential fit for complex **1** (“t1” lifetime in µs) in PLIM (selected pixels). CHO-K1 cells incubated with DMEM-FBS-PEG(200) solution of complex **1** for 24 h. Hypoxia, temperature 310 K, excitation at 405 nm. Content of DMEM 88.5 % v/v, FBS – 10% v/v, PEG(200) – 1.5% v/v, **1** – 5 µM.

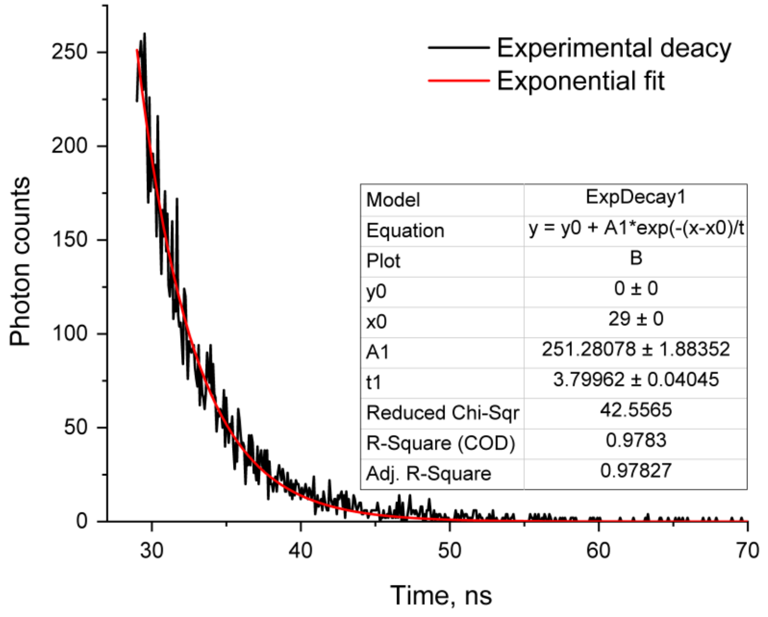


Figure S53. Example of phosphorescence intensity decay curve and its monoexponential fit for complex **7** (“t1” lifetime in μs) in PLIM (selected pixels). CHO-K1 cells incubated with DMEM-FBS-PEG(200) solution of complex **7** for 24 h. Normoxia, temperature 310 K, excitation at 405 nm. Content of DMEM 87.5 % v/v, FBS – 10% v/v, PEG(200) – 2.5% v/v, **7** – 10 μM.

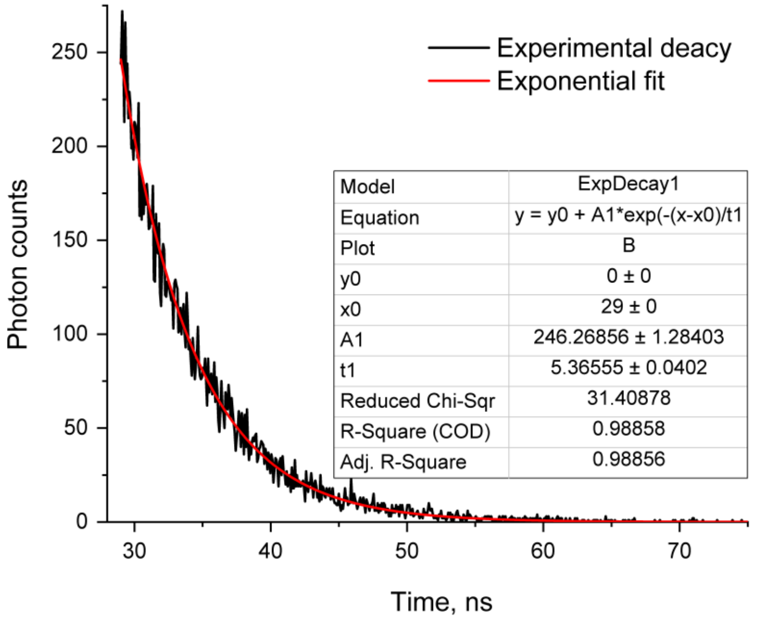


Figure S54. Example of phosphorescence intensity decay curve and its monoexponential fit for complex **7** (“t1” lifetime in μs) in PLIM (selected pixels). CHO-K1 cells incubated with DMEM-FBS-PEG(200) solution of complex **7** for 24 h. Hypoxia, temperature 310 K, excitation at 405 nm. Content of DMEM 87.5 % v/v, FBS – 10% v/v, PEG(200) – 2.5% v/v, **7** – 10 μM.
